# Scalable cell-specific coexpression networks for granular regulatory pattern discovery with NeighbourNet

**DOI:** 10.1101/gr.281171.125

**Published:** 2026-04

**Authors:** Yidi Deng, Jiadong Mao, Jarny Choi, Kim-Anh Lê Cao

**Affiliations:** 1 Melbourne Integrative Genomics, School of Mathematics and Statistics, The University of Melbourne, Parkville, Victoria 3010, Australia;; 2 Research School of Finance, Actuarial Studies & Statistics, The Australian National University, Canberra 2601, Australia;; 3 Bioinformatics and Cellular Genomics, St Vincent’s Institute, Fitzroy, Victoria 3065, Australia

## Abstract

Gene networks provide a fundamental framework for understanding the molecular mechanisms that govern gene expression. Advances in single-cell RNA sequencing (scRNA-seq) have enabled network inference at cellular resolution; however, most existing approaches rely on predefined clusters or cell states, implicitly assuming static regulatory programs and potentially missing subtle, dynamic variation in regulation across individual cells. To address these limitations, we introduce NeighbourNet (NNet), a method that constructs cell-specific coexpression networks. NNet first applies principal component analysis to embed gene expression into a low-dimensional space, followed by local regression within each cell’s *k*-nearest neighborhood (KNN) to quantify coexpression. This approach improves computational efficiency and stabilizes coexpression estimates, mitigating challenges posed by small sample sizes in KNN regression and the inherent noise and sparsity of scRNA-seq data. Beyond coexpression, NNet supports scalable downstream analyses, including (i) clustering and aggregating cell-specific networks into meta-networks that capture primary coexpression patterns and (ii) integrating prior knowledge to annotate coexpression and infer active signaling interactions at the individual cell level. All functional modules of NNet are implemented with an efficient algorithm that enables the application to large-scale single-cell data sets. We demonstrate NNet’s effectiveness through three case studies on transcription factor activity prediction, early hematopoiesis, and tumor microenvironments. Provided as an R package, NNet offers a novel framework for exploring cellular variation in coexpression and integrates seamlessly with existing single-cell analysis workflows.

Gene networks provide essential frameworks for understanding the complex molecular interactions that regulate gene expression in biological systems. At the core of these networks lies the inference of gene regulation, which reflects the binding of transcription factors (TFs) to specific DNA sequences to either activate or repress target gene expression. This regulation directs crucial cellular processes such as differentiation, proliferation, and responses to environmental stimuli. The emergence of high-throughput sequencing, particularly in transcriptomics in the early 2000s, has enabled rapid and affordable quantification of gene expression. Since then, substantial research efforts have been dedicated to developing statistical network inference methods that leverage associations in gene expression (coexpression) to decipher gene regulation (Langfelder and Horvath 2008; Huynh-Thu et al. 2010). In such networks, edges represent measured coexpression between pairs of genes, and when these measurements carry regulatory (causal) implications, significant coexpression can be interpreted as evidence of gene regulation. These advances offer deep insights into cellular function and have contributed to the identification of key regulators of disease development, providing potential avenues for therapeutic intervention.

Single-cell RNA sequencing (scRNA-seq) has provided an unprecedented view of gene regulation by capturing gene expression profiles at the level of individual cells (Badia-i Mompel et al. 2023). Cells are often grouped into clusters based on their expression profiles to represent predefined cell states, and gene networks are inferred by measuring coexpression within these clusters to recover cell state–specific regulatory programs (Chan et al. 2017; Kamimoto et al. 2023; Morabito et al. 2023; Zhang et al. 2023). Although clustering-based network inference methods have been the most widely investigated, they have a major limitation in assuming that cells within a cluster share similar and discrete regulatory programs. This assumption can mask subtle regulatory changes within each cluster and overlook fine-grained regulatory interactions that occur during cell state transitions. In addition, the accuracy of clustering-based methods relies heavily on the accuracy of cell clustering, meaning that poorly defined clusters can lead to biased or oversimplified findings.

To overcome these limitations, researchers have developed cell-specific network (CSN) inference methods that measure coexpression within the local neighborhood of each cell. CSN methods can be broadly classified into two categories based on how neighboring cells are defined. Some methods define neighboring cells based on their similarity in expression profiles (Dai et al. 2019; Wang et al. 2021; Zhang et al. 2022). Coexpression measures in these methods are typically based on correlation, meaning that they do not necessarily indicate gene regulation. Other methods define neighboring cells along a developmental timeline, which can be inferred through pseudotime analysis (Wang et al. 2023; Zhang and Stumpf 2023). In this approach, coexpression measures are time-dependent, potentially capturing causal (regulatory) relationships by associating gene expression at successive points in the trajectory. Despite their advantages, CSN inference faces key challenges in both scalability and stability. Inferring networks for thousands of cells is computationally intensive and often restricted to transcription factor interactions, limiting the biological scope of the networks. The intrinsic sparsity and noise of scRNA-seq data can also compromise the stability of coexpression estimates within small neighborhoods. Moreover, many existing methods lack accessible implementations and offer only limited downstream analyses of inferred networks, hindering their practical application and reducing their impact on biological discovery (Pratapa et al. 2020; Nguyen et al. 2021).

To address these challenges, we present NeighbourNet (NNet), a novel method that uses *k*-nearest neighbor (KNN) principal component (PC) regression on gene expression data to efficiently construct robust, cell-specific coexpression networks. Intuitively, the coexpression between two genes in a given cell is quantified by how strongly one gene embedded within the PCs (i.e., predictor genes) contributes to the local regression model that predicts the expression of the response gene within the cell’s KNN. Our KNN-PC regression approach efficiently captures coexpression at scale. Rather than computing pairwise coexpression, it models a gene’s expression using a small set of PCs, enabling simultaneous estimation of its coexpression with thousands of other genes, hence dramatically reducing computational cost. Embedding in PC space also mitigates the sparsity inherent in single-cell data, yielding more stable and noise-resistant coexpression estimates. Together, these features allow NNet to construct large-scale, cell-specific networks that are both fast to compute and robust. Beyond network construction, NNet is the first framework to provide a comprehensive suite of downstream analyses on CSNs: meta-network analysis to uncover common coexpression patterns, meta-TF analysis to identify gene modules, and prior knowledge integration to facilitate cell-specific inference of active gene regulation and upstream signaling pathways (USPs). NNet, along with its downstream analysis modules, are available as an R package that integrates seamlessly with the widely used Seurat pipeline.

## Results

### Overview of NNet

We begin by outlining the workflow of NeighbourNet (NNet) and introducing the key terminology used in NNet analysis.

#### Coexpression network

**Figure 1.**
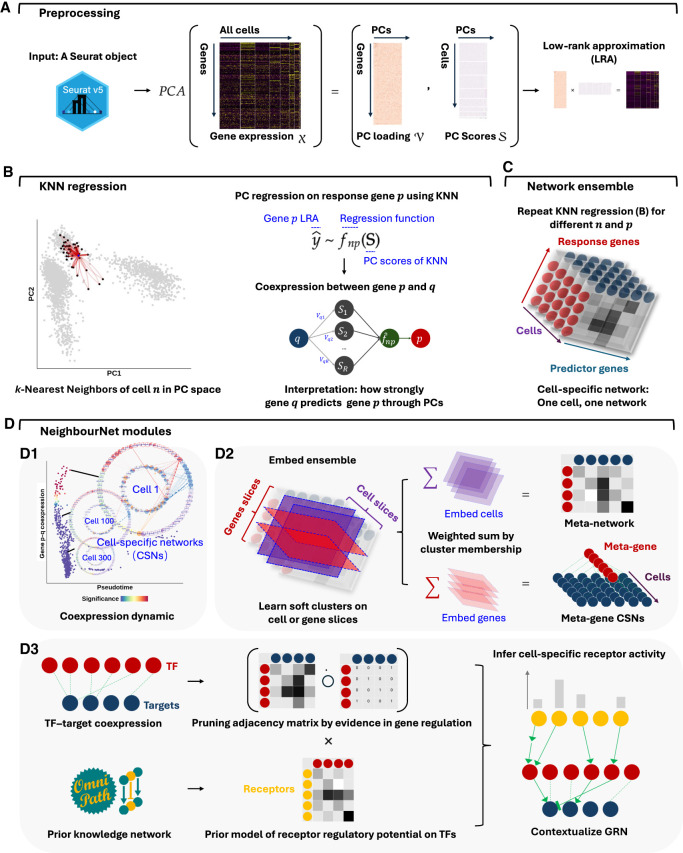
NNet workflow for inferring cell-specific coexpression networks and integrating prior knowledge. (*A*) Preprocessing: Single-cell gene expression data are subject to PCA to capture the major variation. A low-rank approximation (LRA) then reconstructs the expression matrix using the PCs. A weighted *k*-nearest neighbor (KNN) graph that defines each cell’s local neighborhood in the PC space is constructed. (*B*) Neighborhood regression: For each cell, NNet performs regression within the cell’s KNN using the LRA-derived gene expression as the response and PCs as predictors. This process quantifies coexpression between genes by measuring how “predictor genes” (those used to embed PCs) contribute to predict each response in the PC space. Repeating this for multiple response genes yields cell-specific coexpression networks (CSNs). (*C*) Network ensemble construction: Collecting all CSNs form a network ensemble, which is a *cell* × *response* × *predictor* 3D array that can be diced and sliced. (*D*) Downstream analysis: (*D1*) We can slice the network ensemble to examine CSNs, analyzing cellular variation and gene regulation dynamics through coexpression. (*D2*) Nonnegative matrix factorization (NMF) identifies soft clusters of cells with similar network structures. Aggregating CSNs by these clusters yields meta-networks that summarize shared coexpression patterns. Same analysis strategy can be applied to the gene dimension corresponding to TFs, producing TF modules that coregulate common programs. Aggregating TFs by module yields simplified meta-TF–target networks with improved interpretability. (*D3*) We adapted the NicheNet framework to integrate gene regulation and signaling interaction databases from OmniPath, constructing integrated prior knowledge networks (PKNs). Annotating CSNs with these PKNs transforms them into contextualized gene regulatory networks (GRNs). In addition, PKNs enable the inference of upstream signaling pathways (USPs) for each CSN by tracing signal transduction paths (receptor–TF–target) inferred based on the contextualized TF–target interactions.

NNet estimates coexpression at the level of individual cells by combining dimensionality reduction with local regression. To measure cell-specific gene coexpression, NNet first uses principal component analysis (PCA) to embed gene expression data into a low-dimensional space (Fig. 1A). Within this space, each cell finds its *k*-nearest neighbors (KNNs), and a regression model is fitted to predict the expression of a response gene using the PCs. Within the neighborhood of cell *n*, the coexpression level between the response gene *p* and a gene *q* contributing to PC computation (a predictor gene), denoted CSN_*npq*_, is defined as how strongly the gene *q* influences the prediction of *p* through PCs given the fitted model.

By repeating this regression procedure across all response genes, NNet constructs a weighted coexpression network CSN_*n*.._ for every individual cell, represented by a matrix of coexpression values between predictor and response genes for that cell (Figs. 1B,C). As NNet is regression-based, switching the roles of response and predictor genes yields different coexpression values for the same gene pair. The choice of responses depends on context, with the general rule of keeping the number of responses smaller than the number of predictors to reduce computational cost.

#### Embedding network ensemble

The collection of CSNs builds a network ensemble, which is a *cell* × *response* × *predictor* three-dimensional array (a “cube”) where each slice along the cell dimension corresponds to a CSN. While this per-cell resolution is powerful, it can also be helpful to extract higher-level structures that summarize thousands of such CSNs. To achieve this, NNet employs nonnegative matrix factorization (NMF) to identify coherent coexpression patterns across the ensemble, grouping cells that share similar network structures into soft clusters and summarizing them as corresponding meta-networks (Fig. 1D1,2).

Each soft cluster of cells is represented by a matrix H, where each column $H_{.i}$ is a weighting vector defining cluster *i*, and each entry $H_{ni}$ indicates the degree of membership of cell *n* to that cluster. NMF learns these soft clusters based on cell–cell similarity in their networks, and the corresponding meta-network for cluster *i* is computed as a weighted average of the individual CSNs:

$${\text{Meta-Network}}_{i}=\sum_{n}{\text{CSN}}_{n..},H_{ni}.$$

Our NMF approach extracts multiple soft clusters and meta-networks in sequential order, such that meta-network 1 captures the most significant coexpression pattern present in the data set.

The same strategy can be applied to learn soft clusters of genes, summarizing slices of the network ensemble along either the response or predictor dimension. When applied to TFs, each soft cluster represents a TF module, which is a group of TFs that act together to regulate a common transcriptional program. Aggregating TFs according to their learned module yields meta-TF–target CSNs with only a few meta-TFs.

#### Signaling inference

In parallel, we incorporate prior knowledge by mapping observed coexpression edges to curated regulatory and signaling interaction databases. This step produces context-specific gene regulatory networks (GRNs) with regulatory directories and allows for the inference of upstream signaling pathways (USPs) that connect ligand–receptor interactions to transcriptional responses through contextualized TF–target coexpression (Fig. 1D3). The output includes interpretable networks, tools for visual exploration of networks’ regulatory structures, and quantification of active receptor signaling across individual cells.

### NNet coexpression profiles between TFs and targets provide robust evidence for detecting active gene regulation

**Figure 2.**
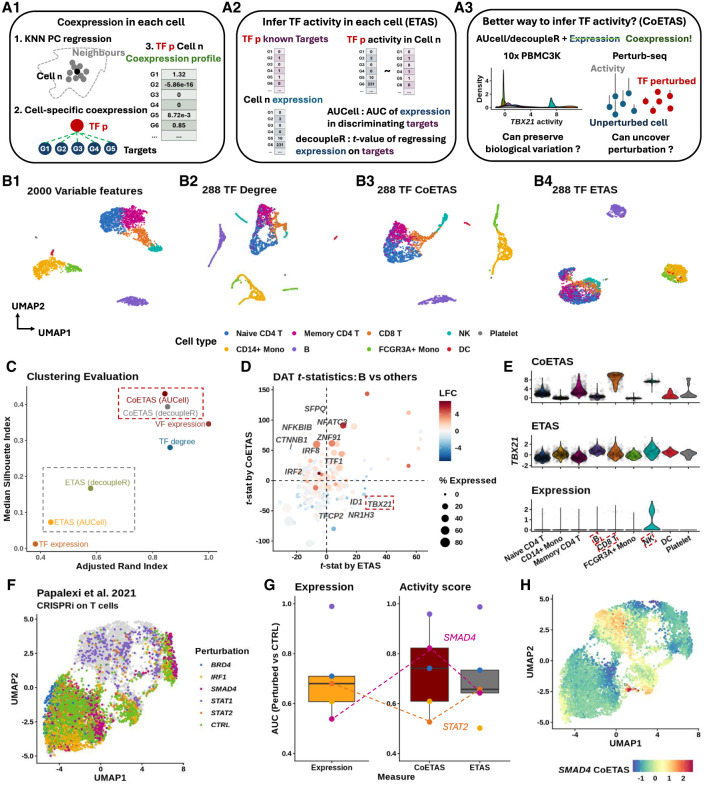
Coexpression between transcriptional factors and targets provides robust evidence to active gene regulation. (*A*) (*A1*) NNet measures the coexpression between each TF and its potential targets in each cell’s neighborhood, generating a TF–target coexpression profile. These profiles serve as input for TF activity inference. (*A2*) Traditional methods such as AUCell and decoupleR typically use target gene expression to infer TF activity, which can be misleading if high expression does not reflect active regulation. (*A3*) With NNet, AUCell or decoupleR are instead applied to coexpression profiles rather than expression profiles. This approach is expected to yield activity scores that better reflect true TF regulation. We benchmarked all approaches by comparing combinations of input (expression vs. coexpression) and method (AUCell vs. decoupleR). (*B*) UMAP visualization of PBMC 3K data. Cells were embedded with (*B1*) the 2000 most variable features (VFs), (*B2*) node degree for 288 TFs in cell-specific networks, (*B3* vs. *B4*) TF activity inferred from coexpression (CoETAS) versus expression (ETAS) using decoupleR. CoETAS preserves cell-type boundaries more effectively, resulting in clearer clustering. (*C*) The adjusted Rand and silhouette indexes were used to quantify clustering quality based on TF activity scores. Including the 2000 VFs and 288 TF expression as controls confirms CoETAS’s superior clustering performance, matching (*B3*). (*D*) *t*-tests on decoupleR-derived CoETAS and ETAS identified differentially activated TFs. The *x*- and *y*-axes show *t*-statistics from ETAS and CoETAS, respectively. TFs are colored by their log-fold change expression in B cells versus other cell types and sized by the percentage of B cells expressing the TF. Many TFs marked as active by ETAS alone (e.g., *TBX21*: a T/NK cell regulator) have low expression in B cells, suggesting false positives. (*E*) Comparing *TBX21* activity across cell types shows that ETAS (*top*) mistakenly marks *TBX21* as active in B cells, whereas CoETAS (*middle*) correctly highlights its known role in T/NK cells. The *bottom* panel shows *TBX21*’s log-normalized expression. (*F*) UMAP of the Perturb-seq data set from Papalexi et al. (2021), with cells carrying TF perturbations highlighted. (*G*) For 5 TF perturbations, ETAS and CoETAS were computed using decoupleR and evaluated by their ability to distinguish perturbed cells from controls using area under the curve (AUC). TF expression was served as a baseline. On average, CoETAS outperformed both ETAS and the baseline, with a particularly strong improvement for *SMAD4*. CoETAS performed poorly for *STAT2*, whose perturbed cells show little separation from controls in *F*. (*H*) CoETAS scores for *SMAD4* show a distinct activity drop among *SMAD4*-perturbed cells.

NNet introduces a novel framework for measuring cell-specific coexpression (Fig. 2A1). To evaluate the quality of the coexpression estimates obtained from PC regression and their relevance for gene regulation, we tested whether TF activity could be accurately inferred using NNet-derived TF–target coexpression profiles. Although NNet does not calculate TF activity itself, these profiles can be used as input for established inference tools, namely AUCell and decoupleR (Aibar et al. 2017; Badia-i Mompel et al. 2022).

Traditional TF activity analysis, including AUCell and decoupleR, takes single-cell expression data as input and produces activity scores for each TF in each cell. For a given TF, the inference is based solely on the expression levels of its known targets: if a cell exhibits higher expression of those targets relative to other genes, the cell is assigned a higher activity score on that TF (Fig. 2A2). However, high target expression does not necessarily indicate active TF regulation, often leading to false positives. Importantly, the same inference tools can also take cell-specific TF–target coexpression as input. By replacing target expression with coexpression profiles, NNet increases the robustness of TF activity inference: activity is now assessed by testing whether a TF is strongly coexpressed with its known targets within each cell’s neighborhood, which is a signal more likely to reflect genuine regulatory activity (Fig. 2A3).

We hypothesised that coexpression–based inference should carry stronger regulatory relevance than expression alone, and therefore produce TF activity scores that more accurately capture true cellular states. To validate this, we benchmarked activity scores derived from expression (ETAS, expression-based TF activity scores) against those derived from coexpression (CoETAS, coexpression-based TF activity scores).

#### Data and setting

We analyzed two data sets. The first was the PBMC 3K data set from 10x Genomics, often used as a benchmark. The second data set was a Perturb-seq experiment (Papalexi et al. 2021), in which the THP-1 monocyte cell line was subjected to pooled CRISPR screening to characterize the molecular networks regulating PD-L1 expression (Table 1).

**Table 1.** NeighbourNet regression setting and computational cost

Data	Cells (*N*)	Responses (*P*)	Predictors (*Q*)	Computational time (s)	Memory usage (GB)
PBMC3k data	*N* = 2638	*P* = 288	*Q* = 4116	LVC: 34.56	25.60
Case study 1		CollecTRI TFs	CollecTRI targets	Regression: 784.52	
Perturb-seq data 1	*N* = 7411	*P* = 5	*Q* = 5151	LVC: 155.39	9.35
Case study 1		CollecTRI TFs	CollecTRI targets	Regression: 18.6	
(Papalexi et al. 2021)					
Perturb-seq data 2 (d7)	*N* = 16,506	*P* = 10	*Q* = 3227	LVC: 872.87	5.62
Supplemental Results		perturbed TFs	CollecTRI targets	Regression: 120.48	
(Dixit et al. 2016)					
Perturb-seq data 2 (d13)	*N* = 9633	*P* = 10	*Q* = 3227	LVC: 305.20	3.00
Supplemental Results		perturbed TFs	CollecTRI targets	Regression: 63.18	
(Dixit et al. 2016)					
Lin^−^ early hematopetic cell atlas	*N* = 1078	*P* = 805	*Q* = 4600	LVC: 33.68	
Case study 2	(Subsampled)	PKN TFs	PKN targets	Regression: 1045.09	34.50
(Pellin et al. 2019)				Meta-network 482.11	
Small cell lung cancer atlas	*N* = 2909	*P* = 28	*Q* = 900	LVC: 87.03	
Case study 3	(Subsampled)	DEGs	PKN TFs	Regression: 66.61	1.23
(Chan et al. 2021)				Meta-network 39.11	

We summarize the number of cells (*N*), the number of response genes (*P*), and the number of predictor genes (*Q*) involved in the NNet analysis for each case study. Each NNet analysis thus generates an ensemble of *N* × *P* × *Q* coexpression networks. For NNet analyses in the early hematopoiesis (Case study 2) and lung cancer (Case study 3) case studies, networks were built on a subset of cells (indicated by “subsampled”) to represent the full data set, further reducing computational burden. The Computational time column records the runtime of NNet in seconds, broken down into the stages of local gene variance calculation (LVC), regression, and meta-network construction. LVC calculation is an initial part of the coexpression measurement (Methods) and only needs to be performed once before regression. Adjusting the response genes for subsequent regression steps does not require recalculating local variance. The Memory usage column shows the change in memory (in gigabytes) before and after the LVC and regression steps. All the analysis were ran on a RStudio server allocated with four cores of Intel Xeon Gold 6254 @ 3.10 GHz CPU. We did not perform parallel computing. Other acronyms: DEGs: differentially expressed genes. PKN: prior knowledge network.

For the PBMC data set, our aim was to perform a sanity check to assess whether TF activity inference based on NNet-derived coexpression is biologically meaningful. Specifically, we analyzed 288 TFs with sufficient target coverage according to the CollecTRI gene regulation database (Müller-Dott et al. 2023) (Methods). Using NNet, we computed each TF’s coexpression with 4116 potential target genes (i.e., any gene with a known TF regulator in CollecTRI). From these coexpression profiles, we derived CoETAS and compared them with ETAS generated directly from target gene expression. We then evaluated which scores better preserved cell-type clustering and better highlighted known cell-type-specific TFs. This comparison serves as a validation step, showing that NNet’s coexpression measure provides sufficient biological signal to capture meaningful regulatory variation.

For the Perturb-seq data set, which includes perturbations on 5 TFs across different subsets of cells, we again used NNet to measure coexpression between perturbed TFs and 5151 target genes. Subsequently, we calculated ETAS and CoETAS for each of the 5 perturbed TFs across all cells. Because the data set provides precise information on TF perturbations in each cell, it allowed us to directly assess how well the inferred activity scores distinguish perturbed from unperturbed cells. This, in turn, provides a hint of whether NNet’s coexpression measure carries accurate information about TF regulatory status.

#### NNet coexpression-based TF activity scores better preserves cell clusters in PBMC data

We annotated the data set by first performing cell clustering based on the expression of the 2000 most variable genes, followed by cell type assignment using cluster-specific marker genes (Fig. 2B1). Using NNet, we constructed CSNs, which enabled novel analyses of cellular variation based on network topology, such as connectivity (i.e., node degree). A UMAP embedding of cells based on the connectivity profiles of 288 TFs within these networks largely preserved the separation of cell types (Fig. 2B2).

Using both target gene expression and NNet TF–target coexpression, we computed TF activity scores and assessed how well these scores preserve cell type clusters. UMAP embeddings showed that CoETAS (Fig. 2B3) better captured known cell types compared to ETAS (Fig. 2B4, Supplemental Fig. S1A). Quantitative evaluations using the adjusted Rand index and the silhouette index further confirmed that CoETAS produced higher-quality clustering (Fig. 2C). As a control, clustering based on TF expression performed worse than TF activity. Additional evaluations using clusters derived from low-rank approximations of TF expression (Supplemental Fig. S1B) ruled out the possibility that the advantage of CoETAS stemmed from the PCA regression used to measure coexpression. These results highlight the ability of NNet coexpression in capturing cell-specific regulatory activity.

#### NNet coexpression profiles detect more meaningful cell-type-specific gene regulation in PBMC data

We then assessed the biological relevance of differentially activated TFs (DATs) identified by activity scores in different cell type clusters. We focused on B cells, which formed the most distinct cluster within the PBMC data (Fig. 2B). We conducted *t*-tests on activity scores to extract DATs in B cells. The *t*-tests on ETAS and CoETAS revealed largely different sets of DATs (Fig. 2D). Whereas most DATs identified by both methods were differentially expressed by B cells, several DATs identified solely by ETAS were actually downregulated or not expressed at all by the B cell population. A key example was *TBX21*, a master regulator of T and NK cells (Szabo et al. 2000; Knox et al. 2019), which was deemed active in B cells by ETAS. In contrast, CoETAS correctly highlighted the relevance of *TBX21* in T cells and NK cells (Fig. 2E). Thus, relying solely on target expression for TF activity inference can yield spurious results, whereas NNet coexpression provides more accurate evidence for genuine regulatory activity.

#### NNet coexpression-based TF activity scores distinguish perturbation effects more effectively in the Perturb-seq data

We further assessed the robustness of CoETAS by analyzing TF perturbation (knockdown) experiments in the THP-1 cell line. A UMAP embedding of cells showed variable responses to five TF perturbations, with *STAT1* knockdown producing the strongest effect, as perturbed cells were clearly separated from the control cluster (Fig. 2F). For each perturbed TF, we computed CoETAS and ETAS using decoupleR, and used area under the curve (AUC) to evaluate their ability to distinguish perturbed cells (expected lower activity; negative class) from controls (expected higher activity; positive class). On average, CoETAS outperformed both ETAS and raw TF expression, achieving higher AUC scores (Fig. 2G). The largest performance gain was observed for *SMAD4*, where CoETAS clearly identified reduced activity in *SMAD4*-perturbed cells (Fig. 2H), whereas ETAS patterns were less distinct (Supplemental Fig. S1C1). In contrast, CoETAS performed worst for *STAT2*, which is likely due to the weak perturbation effect suggested by the close proximity of *STAT2*-perturbed cells to controls in the UMAP. Nevertheless, CoETAS still highlighted a subcluster enriched for *STAT2*-perturbed cells (Supplemental Fig. S1C2).

We performed a similar analysis on an independent Perturb-seq data set (Dixit et al. 2016) containing more TF perturbations (Supplemental Results; Supplemental Fig. S1) and confirmed the advantage of CoETAS over ETAS in detecting perturbation effects.

Overall, our findings show that incorporating NNet coexpression significantly enhances the accuracy and biological relevance of TF activity inference, underscoring its potential in effectively capturing gene regulation activities. Additional analysis comparing NNet with two other cell-specific methods, oCSN (Dai et al. 2019) and LocCSN (Wang et al. 2021), revealed that NNet substantially improves CSN construction by better preserving cell-type identity (Supplemental Results; Supplemental Fig. S7).

### NNet cell-specific coexpression networks enable granular profiling of dynamic shifts in gene regulation

**Figure 3.**
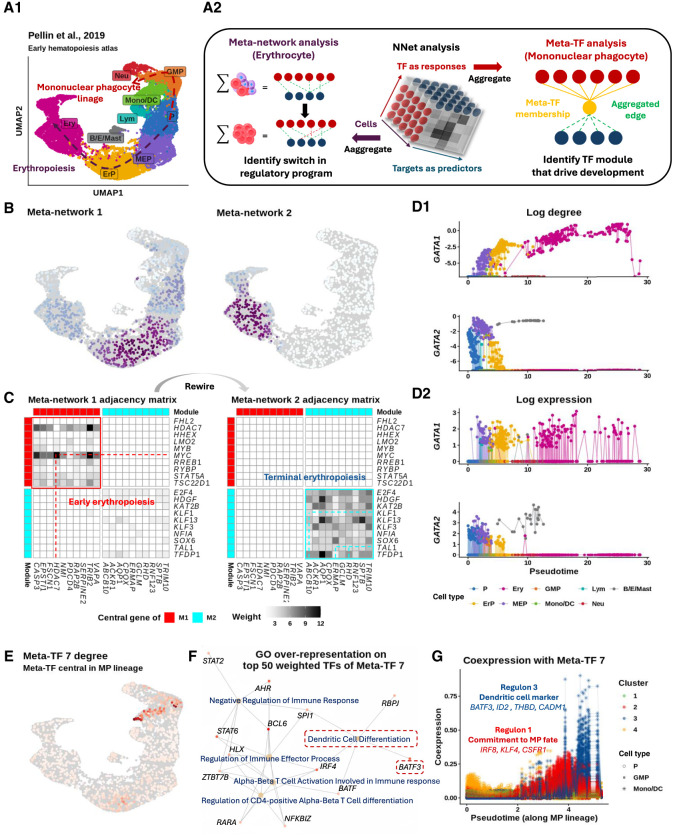
Dynamic coexpression network reveal key transcriptional program shifts during early hematopoiesis. (*A*) UMAP visualization of bone marrow Lin^−^ cells, depicting the transcriptional landscape of early hematopoiesis (Pellin et al. 2019). P: progenitor, GMP: granulocyte-monocyte progenitor, MP: mononuclear phagocyte, Neu: neutrophil, Lym: lymphoid cell, Mast: Mast cell, MEP: megakaryocyte–erythroid progenitor, ErP: erythroid progenitor, Ery: erythrocyte. (*B*) Bipartite coexpression networks were constructed for individual cells, linking TFs to target genes. Nonnegative matrix factorization (NMF) was applied to (i) embed the cell dimension into meta-networks and (ii) or to embed the TF dimension into meta-TFs. Meta-network and meta-TF analyses were used to resolve erythropoiesis (*B*,*C*) and mononuclear phagocyte (MP) differentiation (*E*–*G*). (*B*) Meta-network analysis assigns cell weights that define soft clusters of cells based on their coexpression patterns. Projecting the soft clusters onto a UMAP shows that the first two meta-networks capture major transcriptional program shifts during erythropoiesis. (*C*) Aggregating CSNs according to the soft clusters in *B* yields meta-networks 1/2 (early/late erythropoiesis) representing the clusters. Weighted adjacency matrices of meta-networks are shown as heatmaps. Top 10 TFs (rows) and targets (columns) from each meta-network reveal rewiring from a stemness-associated state (meta-network 1) to erythrocyte-autonomous programs (meta-network 2), indicating an abrupt transcriptional transition. (*D*) Continuous tracking of GATA gene (GATA1 and GATA2) regulatory dynamics during erythropoiesis. (*D1*) tracks changes in connectivity (log node degree) for GATA genes within CSNs during erythropoiesis. Compared to the gene expression patterns shown in (*D2*), connectivity provides a more precise representation of the regulatory dynamics of these TFs, highlighting a distinct switching behavior between GATA2 and GATA1. (*E*) Meta-TF analysis assigns TF weights that define modules (i.e., soft clusters) of TFs based on their coexpression patterns. Aggregating TFs according to these modules yields simplified CSNs that summarize meta-TF–target coexpression. Degree of meta-TF 7 across cells were projected onto the UMAP. Meta-TF 7 emerged as the first module associated with the MP lineage, displaying the highest connectivity within GMPs. (*F*) Over-representation analysis of the top 50 TFs that composes meta-TF 7 identifies immune differentiation pathways. TFs linked to the enriched terms are colored based on their weights on meta-TF 7. (*G*) Meta-TF 7’s coexpression with each target gene along pseudotime. Each scatter path represents a target. Targets were clustered into four groups based on their coexpression patterns, with clusters 1 and 3 showing staggered activation waves linked to MP commitment and CD141^+^ dendritic cell maturation.

Using a stem cell biology case study, we demonstrate that NNet resolves cellular variation at the level of cell-specific coexpression (Fig. 3A1). We propose an efficient matrix factorization technique to summarize and interpret large-scale coexpression data as meta-networks and meta-genes, revealing overarching coexpression patterns and principal regulatory programs (Fig. 3A2). We demonstrate that the NNet framework facilitates accurate identification of pivotal points in cell fate decisions and enhances our understanding of the underlying transcriptional mechanisms that coordinate these decisions.

#### Data and setting

We used the lineage-negative (Lin^−^) bone marrow cell scRNA-seq data from (Pellin et al. 2019), which was designed for profiling the early human hematopoietic landscape (Table 1). With TF and target gene sets acquired from our integrated prior knowledge network (PKN) of gene regulation (Methods), NNet analysis was performed to measure coexpression between 805 TFs and 4600 potential targets.

In the following sections, we perform meta-network analysis and meta-TF analysis separately to study two distinct major lineages: the erythroid lineage and the mononuclear phagocyte (MP) lineage. Meta-network analysis is used to examine transcriptional program alterations during erythropoiesis, whereas meta-TF analysis is applied to identify the key TF module that potentially coordinates MP differentiation.

#### NNet meta-network analysis reveals abrupt rewiring of coexpression network during erythrocyte maturation

We identified 20 meta-networks, each characterized by a set of cell weights indicating the cell populations (i.e., soft cluster) they represent (Supplemental Fig. S2A; see Methods for a detailed definition of cell weights). These meta-networks captured key transcriptional shifts during hematopoiesis. Specifically, the first four meta-networks delineated the primary myelopoietic lineages towards erythroid (Fig. 3B) and MP fates (Supplemental Fig. S2B,C). For example, meta-network 1, primarily composed of networks of megakaryocyte–erythroid progenitors (MEPs), was associated with early lineage commitment characterized by *HDAC7*-driven suppression of *MYC* that releases erythroid differentiation blockage (Fig. 3C) (Delgado and León 2010; Jayapal et al. 2010; Wang et al. 2020). In contrast, meta-network 2, which exhibited increased involvement during terminal erythropoiesis, highlighted a shift towards network modules related to hemoglobin synthesis (e.g., *KLF1*-mediated *ABCB10* expression) (Tallack et al. 2010) and erythrocyte membrane protein synthesis (e.g., *TAL1*-mediated *RHD* and *ERMAP* expression) (Kassouf et al. 2010). Furthermore, the cell weights on meta-network 2 sharply localized to erythroid progenitors (ErPs) from MEPs, suggesting that erythrocyte maturation may involve abrupt, temporally distinct transcriptional reprogramming. Our results recapitulate a well-established TF regulation mechanism known as the “GATA switch,” in which the repression of *GATA2* facilitates a surge in *GATA1* expression: a crucial step in erythrocyte maturation (Suzuki et al. 2013; Bresnick et al. 2018).

#### NNet network topology of individual cells better characterizes temporal dynamics of GATA regulation compared to gene expression

Focusing on the GATA genes (*GATA1* and *GATA2*), we explore how NNet network topology analysis provides an enhanced characterization of dynamics of transcriptional program compared to conventional gene expression analysis. We calculated the connectivity of GATA genes within CSNs and plotted this connectivity against the diffusion pseudotime of cells (Fig. 3D1) (Haghverdi et al. 2016). This analysis showed that *GATA2* reached its peak connectivity at the MEP stage, which then sharply declined following the transition to the ErP stage. As *GATA2*’s connectivity dropped to zero, *GATA1* began to show a significant increase in connectivity throughout maturation, emphasizing a critical temporal coordination of *GATA2* and *GATA1* during erythropoiesis. In contrast, plotting the expression of GATA genes against pseudotime lacks critical details about the dynamics of GATA regulation, such as the peak of activity and the switching point between transcriptional states (Fig. 3D2).

#### NNet meta-TF analysis identifies key transcriptional programs that potentially direct CD141^+^ dendritic cell differentiation

We proceed to illustrate the power of meta-TF analysis, focusing on the identification of key meta-TFs and their regulons associated with MP differentiation, a process distinct from erythropoiesis. Each meta-TF represents a TF module (i.e., a soft cluster) defined by a set of TF weights. Although analyzing a single TF’s coexpression often provides only a partial view of its function, interpreting TFs within the context of their modules offers an integrative view of their role as a regulatory program.

In each CSN, TFs are aggregated according to modules to analyze meta-TF level coexpression with targets (Supplemental Fig. S2D). By examining the connectivity of meta-TFs within each cell, we identified meta-TF 7 as the first one that showed strong connectivity across the MP lineage, particularly in granulocyte–monocyte progenitors (GMPs) (Fig. 3E; Supplemental Fig. S2E). This suggests a potential role for meta-TF 7 in MP differentiation. To functionally characterize meta-TF 7, we performed a Gene Ontology (GO) over-representation analysis on its module composition. This analysis revealed that meta-TF 7 was closely associated with immune cell differentiation (Fig. 3F), particularly the differentiation of dendritic cells (DCs), which belong to the MP lineage. Notably, the DC differentiation term was enriched by *BATF3*, a key TF that is essential for the development of conventional type 1 DCs (cDC1) (Hildner et al. 2008; Satpathy et al. 2012). This clinically significant DC subtype plays a critical role in anti-cancer immunity (Böttcher et al. 2018), with extensive research dedicated to developing protocols for its in vitro differentiation (Elahi et al. 2022; Rosa et al. 2022). We then sought to identify and comprehend potential downstream targets of meta-TF 7 to gain a deeper understanding of its function. Figure 3G illustrates the changes in target gene coexpression with meta-TF 7 during MP differentiation. By clustering targets based on these coexpression values, we identified two major regulons that exhibited staggered waves of activity (Supplemental Fig. S2F).

The first wave of activity, represented by regulon 1, included *IRF8*, *KLF4*, and *CSF1R*, which are critical markers indicative of MP fate commitment (Kurotaki et al. 2014; Tussiwand and Gautier 2015; Combes et al. 2021). The second wave, represented by regulon 4, featured *BATF3* alongside other key markers of cDC1 identity, including *ID2*, a TF that delineates the cDC1 lineage (Jackson et al. 2011); *THBD*, which encodes the CD141 surface marker (Anastasiou et al. 2012); and *CADM1*, which is expressed exclusively by cDC1 (Collin and Bigley 2018). These findings further support the role of meta-TF 7 in coordinating the transcriptional programs driving cDC1 differentiation. Notably, our analysis of marker expression revealed that cDC1 constituted only a small population at the tip of the MP lineage, making it challenging to identify through clustering analysis alone. This underscores the effectiveness of our meta-TF analysis in disentangling relevant TF-target interactions within rare cell populations.

In conclusion, our analysis of the early hematopoiesis atlas demonstrates NNet’s capability to analyze cellular variation through coexpression, particularly providing insights into the dynamics of gene regulation throughout cell differentiation. By meta-network (meta-TF) analysis, we successfully identified principal coexpression patterns, TF modules and regulons at the individual cell level without the need for cell clustering.

### NNet effectively integrates prior knowledge to facilitate cell-specific inference of upstream gene regulation signals

Coexpression between genes does not necessarily imply causal relationships in gene regulation. To enhance the interpretability of coexpression networks, we extend NNet to annotate TF–target coexpression by leveraging prior knowledge of gene regulation and signaling interactions. This approach helps us discern active gene regulation from confounding effects. In addition to identifying regulatory relationships, we infer upstream signaling pathways (USPs), the intracellular signal transduction events that start from receptors and lead to the TF-mediated gene regulation. Moreover, as NNet generates CSNs, the NNet USP inference can also be conducted at the individual cell level, allowing us to further infer signaling dynamics that contribute to cell state transitions.

**Figure 4.**
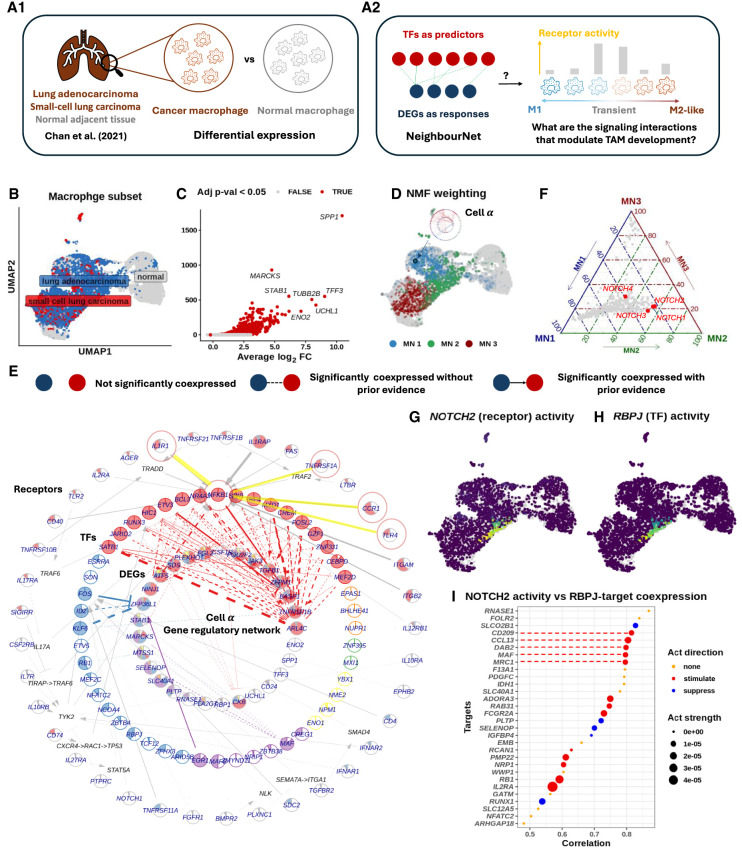
Prior knowledge annotation highlights critical signaling interactions facilitate tumor-associated macrophage development. (*A*) (*A1*) We focused on the macrophage subset of Chan et al. (2021)’s small cell lung cancer scRNA-seq data set. Using NNet, we aimed to identify the signaling interactions that regulate differentially expressed genes (DEGs) between cancer and normal macrophages, providing insights into how inter- and intracellular signaling interactions shape macrophage identity within the tumor microenvironment. (*A2*) We utilized NNet’s upstream signaling pathway (USP) inference to identify receptors with high activity in driving TF regulation of DEGs in transient macrophage populations during TAM development. (*B*) UMAP visualization of macrophages in the data set. (*C*) Volcano plot of the DE result. Only genes that were upregulated in cancer are shown. Pi value: negative log_10_ p-value times log_2_ fold change. (*D*) Soft clusters of cells for the first three meta-networks, illustrating a key transition from proinflammatory macrophages to TAMs. Cell α, with the highest cell weight on meta-network 1, is highlighted. (*E*) Annotated coexpression network of cell α. The *innermost* layer contains response (target) genes, encircled by central TFs in the meta-networks. Receptors occupy the *outermost* layer, each linked to a TF inferred to mediate that receptor’s influence. If the receptor–TF link is indirect, an extra layer shows the shortest signaling path according to prior knowledge. The Methods section provides details. This network captures the canonical NF-kB pathway in proinflammatory macrophages. (*F*) Ternary plot of receptor activity scores on the first three meta-networks. NOTCH receptors (*NOTCH2/3/4*) show peak activity in meta-network 2. (*G*) *NOTCH2* activity and (*H*) *RBPJ* connectivity projected onto the UMAP. *RBPJ* was the TF whose connectivity was found mostly correlated with *NOTCH2* activity. (*I*) Correlation between *NOTCH2* activity and different targets’ coexpression with *RBPJ*. The top targets with the highest correlations are illustrated. *NOTCH2* activity is strongly associated with M2-like TAM marker expression mediated by *RBPJ*.

For our third case study, we analyze scRNA-seq data of small cell lung cancer (SCLC), with a specific focus on its macrophage subset (Fig. 4A1). We demonstrate how NNet with prior knowledge annotation comprehensively reveals critical signal interactions within the tumor microenvironment (TME), which may contribute to the development of tumor-associated macrophage (TAM) phenotypes (Fig. 4A2).

#### Data and setting

We acquired the SCLC atlas from Chan et al. (2021), which includes scRNA-seq data of both tumor and tumor-adjacent normal tissues (Supplemental Fig. S3A; Table 1). Focusing on the macrophage subset (Fig. 4B), we conducted a differential expression (DE) analysis comparing macrophages from tumor and normal samples. Among the top 50 genes upregulated by tumor macrophages, we identified 29 genes that are targets of known TFs according to our PKN of gene regulation (Fig. 4C). The coexpression of these 29 genes with 900 TFs obtained from the PKN was then measured using NNet. We applied meta-network analysis to recover the major coexpression patterns and subsequently utilized USP inference to investigate how signals are transmitted to the 29 genes through TFs across different macrophage landscapes.

#### NNet annotated coexpression networks capture key signaling pathways defining macrophage interactions within TME

We focused on the top three meta-networks and projected the soft clusters they represent onto a UMAP embedding of macrophages (Fig. 4D). These soft clusters distinctly localized to different macrophage populations, suggesting that each population is governed by a unique transcriptional program. To further explore signaling differences among these populations, we visualized the annotated coexpression networks of the three most representative cells (those with the highest cell weight on each meta-network), highlighting a spectrum of macrophage identities (Supplemental Fig. S3B).

On one end of the spectrum, cell *α*, representing meta-network 1, showed a high level of connection across diverse regulatory modules (Fig. 4E). The USP analysis revealed an enrichment of proinflammatory signaling in cell *α*, with *NFKB1* acting as the central TF that mediated signals from receptors like IL1R (*IL1R1*), TNFR1 (*TNFRSF1A*), and TLR4 (*TLR4*). This observation suggests the activation of the well-known NF-kB axis, which strongly supports the M1 proinflammatory identity (as opposed to the M2 anti-inflammatory identity) of cell *α* (Hagemann et al. 2009; Yu et al. 2020).

On the other end of the spectrum, cell *γ*, representing meta-network 3, displayed a complete loss of proinflammatory signaling (Supplemental Fig. S3C1). Instead, it established *ENO1*-mediated signaling to stimulate *SPP1* expression, with SRB1 (*SCARB1*) as the primary receptor and *SRC* likely acting as the transducer relaying the signal to *ENO1*. *SPP1*^+^ macrophages represent a distinct subtype of TAM that has been reported to interact with fibroblasts and anti-inflammatory CD8^+^ T cells, driving tumor growth, metastasis, and immunosuppression (Qi et al. 2022; Bill et al. 2023). While the roles of SRB1, *SRC*, and *ENO1* in TAM have been independently reported, their interaction in promoting *SPP1* expression presents a potential regulatory mechanism that warrants further investigation (Dwyer et al. 2017; Plebanek et al. 2018; Liang et al. 2023).

In between cell *α* and *γ*, cell *β*, representing meta-network 2, exhibited an intermediate transcriptional state with an increase in the activity of the IL4R receptor, which is a critical switch for M2 state activation (Supplemental Fig. S3C2). We hypothesise that cell *β* and the macrophage population associated with meta-network 2 exist in a highly plastic state, rendering them susceptible to reprogramming and valuable targets for therapeutic treatment. To better understand this transitional state, our next analysis investigates the key signaling interactions and their consequences among these transient macrophages in the TME.

#### NNet receptor activity analysis highlights activation of *RBPJ*-mediated NOTCH signaling during transitional macrophage state

NNet’s USP inference involves calculating each receptor’s potential activity across target genes for each cell, summarized as activity scores. Leveraging this inference, we explored the receptors that exhibit differential signaling in transient macrophages, represented by cell *β*, compared to M1 macrophages (e.g., cell *α*) and the TAM population (e.g., cell *γ*). Specifically, we summarized receptor activity within each macrophage population by a weighted sum (using importance scores) of cell-specific receptor activities (Methods). Our comparison revealed that NOTCH (*NOTCH2/3/4*) receptors were exclusively active in the transient macrophage population associated with meta-network 2 (Fig. 4F). Endothelial cells, expressing the NOTCH ligands *DLL* and *JAG*, emerged as the principal source of NOTCH signaling (Supplemental Fig. S3D). This finding aligns with previous research showing that NOTCH signaling can influence macrophage polarization towards the M1 or M2 phenotype, although its precise direction remains a debated topic (Xu et al. 2012; Foldi et al. 2016; Palaga et al. 2018; Chen et al. 2023).

To clarify the role of NOTCH in our lung cancer setting, we examined TF connectivity in relation to *NOTCH2* activity (Fig. 4G). *RBPJ* emerged as the top TF whose connectivity highly correlated with *NOTCH2* activity (Fig. 4H), which is consistent with its established role as a key mediator of NOTCH signaling and a frequent target for blocking the pathway (Friedrich et al. 2022). Our findings partially recapitulate those of Franklin et al. (2014), who reported that *RBPJ*-mediated NOTCH signaling is crucial for the final differentiation of TAMs from tumor-infiltrating monocytes. However, it remains unclear whether this pathway also governs the macrophage transition from the M1 to TAM phenotype. To address this question, we next investigated the downstream targets of *RBPJ* to characterize the functional consequences of NOTCH signaling.

#### RBPJ coexpression pinpoints downstream targets of NOTCH signaling and its role in converting macrophages to TAM

We performed a separate NNet analysis to measure *RBPJ*’s coexpression with 5011 target genes. By calculating the correlation between *NOTCH2* activity (as shown in Fig. 4G) and each target’s coexpression with *RBPJ* across individual cells, we identified a set of genes that were highly associated with NOTCH-RBPJ signaling (Fig. 4I). According to prior knowledge, key genes triggered by this signaling include *MAF* and *DAB2*, which are important regulators of M2 polarization (Liu et al. 2020; Marigo et al. 2020), as well as the cell surface receptors MRC1 (also known as CD206) and CD209, which are defining markers for M2-like TAMs (Wang et al. 2024). Whereas Franklin et al. (2014) suggested that *RBPJ*-dependent TAMs are *MRC1*^−^, our findings indicate that *RBPJ* may induce *MRC1*^+^ TAMs at a transient state. Functionally, we also noted that NOTCH-RBPJ signaling triggered macrophages to secrete chemoattractants such as *CCL13*, which may recruit immune cells to the TME, thereby modulating the immune landscape (Supplemental Fig. S3D).

Taken together, our final case study showcases NNet’s ability to capture signaling interactions that precede gene regulation in individual cells. Through this approach, we identify that NOTCH-RBPJ signaling becomes active at an intermediate macrophage state, positioned between the M1 proinflammatory and M2-like TAM phenotypes. In this transitional phase, NOTCH-RBPJ may modulate re-education signals from the TME, facilitating the conversion of M1 macrophages into TAM.

## Discussion

NNet is a novel and highly scalable framework for constructing cell-specific coexpression networks from scRNA-seq data. Based on PC regression, our approach unravels coexpression within the local neighborhood of individual cells, offering a fresh perspective for understanding regulatory dynamics beyond traditional clustering-based gene network inference methods. NNet is highly scalable, capable of capturing networks for thousands of cells at low computational cost. A key advantage is that it pairs this scalability with accessibility, offering a comprehensive suite of downstream analyses that enables researchers to fully leverage the granularity of cell-specific networks (CSNs) for meaningful biological discovery. We highlighted the versatility of NNet through three case studies and its utility in facilitating the identification of fine-grained regulatory signals pertinent to specific cell states or transitions.

In the first case study of TF activity inference, we performed a proof-of-concept analysis on NNet coexpression. By comparing TF activity scores calculated based on TF–target coexpression with those obtained solely from target expression, we demonstrated that coexpression-based scores more accurately reflect TF function and activation status. These findings showed that NNet coexpression carries reliable evidence of gene regulation and highlight that traditional expression-based TF activity inference may yield higher false discovery rates, warranting more cautious interpretation.

In the second case study on early hematopoiesis, we introduced a novel perspective for analyzing cellular variation through coexpression analysis. We showed that analyses traditionally performed in gene expression space, such as dimensionality reduction, clustering, and pseudotime analysis can be effectively adapted to coexpression space. A key insight from this study was that analyzing coexpression without relying on predefined clusters substantially improves the ability to uncover detailed regulatory signals in scRNA-seq data. The successful identification of the TF module associated with conventional type 1 dendritic cell (cDC1) differentiation exemplified this advantage, as traditional cluster-based approaches tend to obscure features of rare cell populations within broader groupings. Although we do not present results that demonstrate novel biological discoveries, many of our findings warrant further investigation. For example, the composition of the meta-TF module and how its factors co-regulate to promote the differentiation of the clinically significant DC subtype deserve closer study.

In the third case study involving small cell lung cancer, we demonstrate NNet’s capability to integrate prior regulatory knowledge, ranging from gene regulation to signaling interactions, into the functional characterization of coexpression networks. Through comprehensive visualization and quantitative analyses of CSNs, NNet emerges as a pioneering algorithm that resolves fine-grained details of signaling pathways at the individual cell level, addressing a key gap in current computational approaches. We pinpointed a potentially novel role of NOTCH–RBPJ signaling in tumor microenvironment (TME) development through the re-education of M1 proinflammatory macrophages. Franklin et al. (2014) reported that this pathway promotes monocyte differentiation into *MRC1*^−^ tumor-associated macrophages (TAMs). Our results suggest that it may also contribute to the development of *MRC1*^+^ TAMs. However, we cannot draw a definite conclusion about whether the TAMs we analyzed arise from the re-education of existing M1 macrophages or from the primary differentiation of monocytes, simply based on the continuous transition observed between M1 macrophages and TAMs. We also cannot infer that this pathway causally drives the development of *MRC1*^+^ TAMs, and these hypotheses require experimental validation.

We identified some limitations in our study. First, we did not perform simulation-based benchmarks, which are commonly used to validate new method for inferring global or cluster-specific networks. This omission is primarily due to the substantial technical challenges of simulating realistic CSNs. As a result, our validation used real-world data sets and focused on the basic property of CSNs: their ability to preserve first-level cell type information. Second, our downstream network analysis focused on basic network properties such as node degree, primarily to enhance interpretability. Incorporating additional metrics, such as clustering coefficients, modularity, or network motifs, could further illuminate the regulatory complexity and dynamic characteristics of CSN.

Looking ahead, NNet’s framework can be adapted to spatially resolved transcriptomics data, where cell neighborhoods are defined by spatial proximity rather than gene expression similarity. This extension would enable the study of spatial intercellular communication within tissue-specific microenvironments. Furthermore, by adjusting how data embedding and regression are performed within cell neighborhoods, NNet can be applied to infer interactions involving molecules beyond transcripts, including ligand–receptor binding (proteomics) and multiomics relationships. A particularly promising direction is the use of partial least squares (PLS) regression for multiomics embedding, which could facilitate the integration of diverse data modalities and broaden NNet’s applicability across biological contexts.

## Methods

All bioinformatic analyses, including the development of the NeighbourNet software, were conducted in R version 4.5.0 (R Core Team 2025).

### NeighbourNet: cell-specific coexpression network inference

We develop NNet to infer gene networks from scRNA-seq data. Unlike traditional statistical methods that rely on coarse-grained cell type clusters to measure gene coexpression, NNet directly analyses coexpression within each cell’s *k*-nearest neighborhood (KNN) in the gene expression space. To improve computational efficiency and mitigate noise in scRNA-seq data, we apply PCA to embed and denoise gene expression. Coexpression is then measured between PCs and response genes using regression models, and gene-gene coexpression is subsequently recovered from functional dependencies learned between PCs and response genes. In this section, we outline how we build a coexpression network for each cell (cell-specific network, CSN) and how we refine these networks based on the KNN graph of cells, a cell-by-cell matrix describing KNN relationships in gene expression. Briefly, the procedure consists of the following steps:

1. Build a KNN-graph based on PCA, describing neighborhood structure of cells.
2. Fit PC regression within each cell’s neighborhood to build CSN.
3. Smooth the networks using the KNN-graph to reduce noise.
4. Calculate the significance of coexpression based on the KNN graph constructed.

In the following sections, we describe the NNet algorithm in more detail with some mathematical formulation. Additional technical details are provided in Supplemental Methods.

#### Building a KNN graph

We first perform PCA (with centering and scaling) on the *N* × *P* cell-by-gene expression matrix X to obtain an *N* × *R* score matrix S representing *R* principal components (PCs). The choice of *R* follows Linderman et al. (2022) (Supplemental Methods). Using these PCs, we reconstruct a low-rank approximation (LRA) of the expression data $\hat{X}$, which serves as the response variable in PC regression.

Next, we construct a weighted KNN graph of cells represented by an *N* × *N* adjacency matrix *W*. Each cell *n* ∈ {1, 2, …, *N*} in the graph is connected to its neighbors KNN(*n*) based on geodesic distances in the PCA space, following the approach of Becht et al. (2019) (Supplemental Methods). By default, 30 nearest neighbors are considered. Stronger edge weights correspond to shorter distances between cells. See Supplemental Results and Supplemental Figure S5 for an evaluation of tuning parameters.

#### PC regression within each cell’s neighborhood

Within each cell *n*’s neighborhood, we extract the neighboring cells’ LRA and PC scores, denoted by $\hat{X}$ and **S**, respectively (suppressing the index *n* for clarity). For a given gene *p* ∈ {1, 2, …, *P*}, we treat its reconstructed expression $y\equiv {\hat{X}}_{.p}$ as the response, and fit a local PC regression model:

$$y∼f_{np}(S).$$

where cell *m* ∈ KNN(*n*) are weighted in the regression according to their connection strength to *n*, the master cell of the neighborhood, given by $W_{nm}$.

Coexpression levels between the response gene *p* and each predictor gene *q* ∈ {1, 2, …, *P*} contributing to PC computing is defined as permutation feature importance (PFI) score. Because direct permutation of thousands of genes within each local neighborhood is computationally prohibitive, we derive an analytical approximation: PFI is estimated by the dot product between the partial derivatives of ${\hat{f}}_{np}$ with respect to PCs and each gene *q*’s loading vector. If we use a linear model to fit a local PC regression for response gene *p* for cell *n*, with ${\hat{\beta }}_{npr}$ denoting the fitted regression coefficient of PC *r*, then the coexpression between an embedded gene *q* and the response *p* is defined as

$${\text{CSN}}_{npq}=2\mathrm{Var}(X_{.q}){\left(\sum_{r}{\hat{\beta }}_{npr}\frac{V_{qr}}{\left\|V_{q.}\right\|}\right)}^{2}$$

where $\mathrm{Var}(X_{.q})$ is the local variance of gene *q* expression within the neighborhood, and V represents the PC loading matrix.

Repeating this procedure across response genes produces one cell-specific coexpression network per cell, represented by an adjacency matrix:

$${\text{CSN}}_{n}\in R^{P\times P}$$

Our framework supports PC regression with various regression methods, with ridge regression (penalty parameter = 0.5) used as the default. Full derivations and implementation details are provided in Supplemental Methods.

#### Smoothing and significance assessment

Because local regression is performed within relatively small neighborhoods, coexpression estimates may be sensitive to sampling variability. To reduce instability, CSNs are smoothed on the KNN graph of the cells using a random walk operator derived from *W*, which propagates information across neighboring cells while preserving local structure.

Statistical significance of each coexpression edge is then assessed using a neighborhood-agnostic null model. Specifically, we construct a null distribution by recomputing coexpression after shuffling the KNN graph structure, thereby destroying true local dependencies while preserving marginal distributions. This generates a background distribution of smoothed coexpression values under the absence of structured neighborhood effects. For each edge, the observed coexpression is compared against this null distribution, yielding a Wald-type test statistic and a probabilistic significance score between 0 and 1. Users may prune CSNs by applying a significance threshold prior to downstream analyses.

The algorithmic details are provided in Supplemental Methods. An assessment of the proposed significance score is performed on an independent data set from Tian et al. (2019) (Supplemental Results; Supplemental Fig. S6).

#### Restricting NNet analysis to subsampled cells to reduce computational demand

To further reduce the computational demands of NNet, both in terms of time and memory usage, and to make it feasible for use on personal computers, we infer CSNs on a representative subset of cells from the entire data set. To select representative cells, we apply *k*-means clustering to the PCs, setting the number of clusters to match the desired number of representative cells. For each cluster, we select the cell closest to the cluster centroid as the representative.

NNet runs PC regression and build CSNs on the selected representative cells, leveraging their neighboring cells from the complete data set. This yields a smaller network ensemble on which downstream analysis can then be applied. For details on how we perform data smoothing on this network ensemble, refer to Supplemental Methods.

### NNet downstream analysis

Beyond coexpression, NNet supports two major downstream analyses (Fig. 1D). We first provide an overview of the two analyses without diving into technical details.

The first is the use of nonnegative matrix factorization (NMF) to derive soft clusters of cells or genes from the ensemble of CSNs. Soft cell clusters are represented by cell weights, which are used to construct meta-networks via weighted averaging of CSNs, thereby capturing overarching coexpression patterns across groups of cells. Similarly, genes can be clustered and aggregated based on their weights that represent gene modules, creating meta-genes and their coexpression profiles across cells. We optimized the NMF algorithm to make it scales efficiently on the large ensemble we generate.

Second, we integrate prior knowledge to annotate coexpression edges. We construct integrated prior knowledge networks (PKNs) following the NicheNet framework (Browaeys et al. 2020; Müller-Dott et al. 2023), combining curated regulatory and signaling interactions. Coexpression edges supported by prior knowledge are assigned regulatory directionality to generate context-specific gene regulatory networks (GRNs). Building on these networks, we infer upstream signaling pathways (USPs) that connect receptor signal transduction to TF-mediated target regulation. NNet further provides visualization tools to explore inferred gene regulation and signaling interaction.

#### Embedding network ensembles

A three-dimensional network ensemble can be embedded into two-dimensional meta-networks using NMF.

Specifically, each CSN is vectorized into a column of a *P*^2^ × *N* “tall-and-skinny” matrix $A^{\mathrm{cell}}$ where row correspond to edges and columns correspond to individual cells. NMF decomposes this matrix as

$$A^{\mathrm{cell}}=FH^{T}$$

where F is a *P*^2^ × *N*′ factor matrix representing *N*′ vectorized meta-networks, and H is a *N* × *N*′ factor matrix representing *N*′ soft clusters.

To address the computational challenges of applying NMF to large matrices such as $A^{\mathrm{cell}}$, we employ a computationally efficient variant of NMF based on nonnegative principal component analysis (nPCA) (Sigg and Buhmann 2008), a PCA formulation that enforces nonnegative loadings. The computational trick of (Benson et al. 2014) was incorporated into the nPCA algorithm, allowing us to perform factorization on a reduced representation of $A^{\mathrm{cell}}$ without explicitly constructing the full matrix, thereby saving both computation time and memory.

A similar nPCA approach is used for meta-gene (meta-TF) analysis (Case study 2). Instead of retaining the cell dimension, the network ensemble is converted into an *NP* × *P* tall matrix $A^{\mathrm{response}}$ that preserves the response (or predictor) gene dimension. Applying nPCA to this matrix clusters and embeds responses, generating a factor matrix that represent coexpression profile between meta-response and predictors across the cells. Refer to Supplemental Methods for implementation details.

#### Constructing integrated prior knowledge networks

We performed the NicheNet integration of PKN using the R package OmniPath, which not only provides a collection of high-quality databases but also offers a framework for users to choose their own databases with prior knowledge confidence levels (Müller-Dott et al. 2023). See Supplemental Methods for details on our database selection process. We obtained two integrated PKNs as directed graphs:

- **TF–target gene regulatory network**: Comprising 1176 TFs, 6568 targets, and 41,206 gene regulation interactions.
- **Signaling interaction network**: Incorporating 9867 genes with 79,242 signaling interactions.

These PKNs were precomputed and edge-annotated to depict whether they consistently indicate stimulation or inhibition, as aligned in the source databases. Additionally, by executing a personalized PageRank algorithm on the signaling PKN, we precomputed a matrix reflecting the prior regulatory potential of receptors on TFs (Supplemental Methods) (Page et al. 1999; Browaeys et al. 2020):

- **Regulatory potential matrix**: Comprising the regulatory potential of 415 receptors on 905 TFs, aiding in USP inference.

This matrix will be used to facilitate the USP inference.

#### Receptor prioritization

USP inference aims to reconstruct potential intracellular signaling pathways linking receptor activation to TF-mediated regulation of downstream target genes, as reflected by TF–target coexpression in the CSN. USP inference proceeds in two stages. First, we integrate the signaling interaction PKN with CSNs to prioritise receptors based on their potential activity in regulating downstream targets through TFs. Second, for prioritized receptors, we reconstruct the shortest signaling paths linking receptors to TFs using the PKN.

Receptor prioritization follows NicheNet’s ligand activity framework, adapted to our receptor–TF–target setting. Specifically, we compute a receptor–target activity matrix of a cell by taking the dot product between the receptor–TF regulatory potential matrix and the adjacency matrix of a pruned CSN (Fig. 4A2). By summing a receptor’s per-target activity values over all targets, we compute an overall receptor activity score that reflects the strength of the receptor’s regulatory impact on the entire target gene set. Receptors with high activity scores are considered key upstream regulators and are thus prioritized for further exploration. Implementation details are provided in Supplemental Methods.

Intuitively, the receptor activity measures the effectiveness of a two-stage signal transduction: from receptor to TF through the PKN, and from TF to target through coexpression edges in the CSN. Its reliability, therefore, depends critically on the confidence in these edges as proxies for regulatory interactions. To control this, we apply pruning to CSNs prior to downstream analysis at three levels of stringency:

1. **No pruning**: retains all coexpression edges.
2. **Statistical pruning**: removes edges that fail significance testing.
3. **High-confidence pruning (default)**: retains only edges that are both statistically significant and supported by prior regulatory knowledge.

An edge is considered supported if the corresponding TF can transmit regulatory influence to the target within a defined number of steps (two steps by default) in the gene regulation PKN.

#### Reconstructing upstream signaling pathways (USPs)

For each prioritized receptor in each CSN, we first identify TFs that plausibly mediate the signal transduction to targets. Candidate TFs are selected based on having strong node degree in the pruned CSN, and receiving high regulatory potential from the receptor (Supplemental Methods).

For each selected receptor–TF pair, we then use the igraph::shortest_paths function (R package igraph, v2.0.1.1) to identify minimal signaling paths within the signaling interaction PKN, which represents the most direct series of known molecular interactions linking receptor activation to TF modulation. Finally, combining these receptor–TF paths with coexpression-supported TF–target interactions yields receptor–TF–target triplets, representing putative intracellular signaling pathways inferred for each cell.

#### Visualizing prior-knowledge-enriched networks

We visualize prior-knowledge-enriched TF–target coexpression networks using a hierarchical structure, as shown in Fig. 4E.

1. **Target genes**: positioned at the inner most layer.
2. **TFs**: encircle targets, and are grouped by expression-based clustering (node color), assigned significance probabilities (pie charts: % fill = likelihood of coexpression with targets).
3. **USPs**: The outermost layer lays the most active receptors, each connected to a TF that likely mediates the receptor’s influence on the targets via USP inference. Receptors are colored to match TFs, with the percentage of color filling represents the expression level. When a receptor and a TF lack a direct link, USP inference puts an extra layer between them shows their shortest signaling path.

Edges indicate evidence of interaction: none (no edge), significant coexpression only (dashed), or significant coexpression supported by prior evidence (solid, with arrowhead for activation or barhead for repression).

#### Central gene selection

In networks containing thousands of genes, it is difficult to visualize the entire structure, as the dense overlap of edges becomes messy and obscures meaningful information. Therefore, it is often more informative to visualize subnetworks consisting of a subset of central genes (e.g., in Fig. 4E, only central TFs are shown).

We identify central genes by evaluating eigenvector centrality in meta-networks. We apply SVD to each meta-network’s response-by-predictor adjacency matrix. The absolute values of the left and right singular vectors correspond to the centrality of responses and predictors, respectively. Genes with the highest centrality are selected, and the union of top-ranked genes across multiple meta-networks defines the final set of central genes.

### Data for NNet analysis

We provide an overview of the public scRNA-seq data sets utilized in our case study and detail the preprocessing steps performed prior to NNet analysis. All data were preprocessed and analyzed based on a Seurat object in R (Seurat package version: 5.0.3). Unless specifically noted, NNet was configured using the default tuning parameters as described in the Methods section below.

#### The 10x PBMC3k data

We obtained a widely recognised data set of human peripheral blood mononuclear cells (PBMCs), commonly used for benchmarking cell clustering methods, from the Seurat PBMC3k tutorial at https://satijalab.org/seurat/articles/pbmc3k_tutorial (last retrieved: Oct 27th, 2024) (Butler et al. 2018). Following the code outlined in the vignette, we preprocessed the data, performed dimension reduction using PCA and UMAP, and conducted clustering. The resulting data is log-normalized, containing 2638 cells and 13,714 genes.

We treated TFs as responses and target genes as predictors in our NNet analysis. As our TF activity inference relies on gene regulation data from the CollecTRI database (Müller-Dott et al. 2023), we extracted the relevant TF and target gene sets from CollecTRI. Among the 13,714 genes in the data set, 731 TFs and 4116 target genes were identified. To ensure meaningful inference of TF activity, we further filtered the 731 TFs to include only those with at least 10 associated targets according to CollecTRI prior knowledge. This resulted in 288 TFs for NNet analysis on all the cells, maintaining 4116 target genes as predictors.

#### The Perturb-seq data

The data were originally generated by Papalexi et al. (2021), who performed CRISPR screening on the THP-1 monocyte cell line to investigate molecular networks regulating PD-L1 expression in acute leukemia, a potential mechanism of T cell inhibition. The original study perturbed 26 genes that are correlated with PD-L1 expression. We used a cleaned version of this data set obtained from the PerturbSeq.db database (He et al. 2025). The processed data set contains 18,257 genes across 7411 cells, each subjected to one of 10 filtered valid perturbations or left unperturbed as negative controls. Among these 10 genes, 5 are validated TFs: *BRD4*, *IRF1*, *SMAD4*, *STAT1*, and *STAT2*. We did not perform further quality control filtering on genes or cells.

NNet analysis was performed on all cells, using the 5 perturbed TFs as responses. Following the same procedure as with the PBMC data set, we selected 5151 CollecTRI target genes as predictors.

#### The early hematopoiesis atlas

The data generated by Pellin et al. (2019) profiles an early human hematopoietic landscape of bone marrow mononuclear cells that lack mature lineage markers (Lin^−^). We obtained the raw data from the NCBI Gene Expression Omnibus (GEO; https://www.ncbi.nlm.nih.gov/geo/) accession GSE117498 and downloaded the four Lin^−^ samples. These samples were merged into a combined data comprising 24,720 genes and 15,397 cells. Without performing additional quality control to filter out genes or cells, we applied the Seurat pipeline to process the data. We annotated cell clusters according to the marker genes provided by Pellin et al. (2019).

The NNet analysis was conducted on a subset of 1078 cells, using 805 TFs as responses and 4600 targets as predictors. These TF and target gene sets were collected from our PKN with the criterion that they should be expressed by at least 20 cells.

#### The small cell lung cancer atlas

The data set was generated by Chan et al. (2021), who aimed to profile the heterogeneity of small cell lung cancer (SCLC) and its associated microenvironment across different lung cancer subtypes, with a specific focus on identifying and characterizing macrophage subpopulations linked to the presence of a metastatic SCLC subtype. Additionally, they collected and sequenced specimens from lung adenocarcinoma (LUAD) and normal lung tissues for comparative analysis. We downloaded the processed data from the CELLxGENE data portal, which comprises cells pooled from 42 donors. We retained 19,558 genes that have HUGO symbols for easier interpretation and did not perform additional quality control to filter out cells from the processed data. Utilizing the cell type annotations provided by CELLxGENE, we extracted 9251 macrophages, on which we then applied the Seurat pipeline to process the data.

We conducted two separate NNet analyses on the same set of 2909 macrophage that were subsampled: one aimed at identifying signaling interactions associated with protumorigenic macrophage development, and another focused on determining the downstream targets of NOTCH-RBPJ signaling. For the first analysis, response genes were identified through a differential expression (DE) analysis comparing macrophages from tumor and normal samples. From the top 50 differentially expressed genes (DEGs) of tumor macrophages, we selected 28 genes with known TF regulators according to our PKN to serve as responses for NNet analysis. In the second analysis, *RBPJ* was set as the NNet response. When selecting predictors, we only considered genes that were expressed by at least 20 macrophages. As a result, 900 TFs and 5011 targets (not specific to *RBPJ*) in the PKN were selected as predictors for the first and second analyses, respectively.

### AUCell and decoupleR analysis for TF activity inference

The R package decoupleR (Version 2.9.7) provides an ensemble of computational approaches for TF activity inference based on gene expression data (Badia-i Mompel et al. 2022). This ensemble includes the AUCell method and the package’s new approach that relies on univariate linear regression, referred to as the decoupleR method in our paper (Aibar et al. 2017). Both AUCell and the decoupleR method infer TF activity by evaluating how well a cell’s gene expression can predict the known target gene set of a TF. AUCell calculates the area under the curve (AUC) score for the sorted expression of a cell in distinguishing the target gene set. In contrast, the decoupleR method performs a regression between gene expression and a dummy vector representing the target gene set, calculating TF activity as the *t*-value of the regression. Both functions take a gene expression matrix and a prior knowledge GRN as inputs, iterate AUC and *t*-value calculations on each cell for each TF, eventually generating a TF-by-cell matrix of TF activity. The prior knowledge GRN we used was generated by decoupleR::get_collectri based on the CollecTRI database, which was in a specific data format required by the decoupleR functions.

TF activity inference based on TF–target coexpression was also performed using the decoupleR package. The only difference was that, for each TF, we measured its coexpression with targets across cells, generating a TF-specific coexpression profile on which activity inference was then performed.

### Evaluating the effectiveness of TF activity in distinguishing perturbed cells using area under the curve

For the Perturb-Seq data, we used AUCell and decoupleR to measure the activity of 5 TFs that were perturbed. For each of the 5 TFs, we compared the activity scores between two groups of cells: those perturbed exclusively on that TF and those that were not perturbed on any TF (controls). The area under the curve (AUC) was calculated to assess how well the activity score of the perturbed TF could distinguish the unperturbed cells. AUC evaluations for the smaller batch clusters are provided in Supplemental Figure S1. AUC was calculated using the R package pROC (Version 1.18.0) with the function pROC::roc.

### Embedding and clustering PBMC3K data by TF activity

On the PBMC data, we calculated TF activity for 288 TFs across 2638 cells based on different activity inference methods (AUCell and decoupleR) applied to different measurements (expression and coexpression). The resulting TF activity matrices were then embedded using PCA (centred and scaled) followed by UMAP (using 10 PCs).

We employed the adjusted Rand index (ARI) and median silhouette index (MSI) to quantitatively assess how well the activity scores of 288 TFs recapitulate the cell clusters we learnt from the expression of the 2000 most variable features (Rand 1971; Rousseeuw 1987). MSI was calculated based on the distances between cells in the 10 PC space of activity scores, evaluating whether cells within the same gene expression cluster are also grouped together according to their TF activity profiles. ARI is a metric that evaluates the agreement between two different clustering schemes. We utilized ARI to compare clusters generated from activity scores with those derived from gene expression data. For the clustering based on activity scores, we employed Seurat’s clustering pipeline to generate exactly 9 clusters, matching the number of clusters obtained from the expression data.

### Over-representation analysis on meta-TF weighting vectors

We performed an over-representation analysis on the NMF weighting vector of meta-TF 7, the first meta-TF that showed strong connections with target genes in the mononuclear-phagocyte lineage. Using the R package clusterProfiler (version 4.11.1), we identified over-represented Gene Ontology (GO) terms in biological processes among the top 50 weighted TFs of meta-TF 7 (Yu et al. 2012). A false discovery rate threshold of 0.05 was set to determine significant GO terms, and the background gene set for the analysis consisted of the 805 TFs used in the NMF embedding.

The resulting GO terms were ranked according to their pi-values, calculated as the product of their fold changes and their negative log_10_ p-values (Xiao et al. 2014). Highly redundant GO terms were trimmed by the clusterProfiler::simplify function. Finally, the top five nonredundant GO terms with the highest pi-values were illustrated.

### Diffusion pseudotime inference

Using the R package *destiny* (Version 3.10.0), we performed diffusion pseudotime (DPT) inference on the early hematopoiesis atlas to reconstruct the temporal ordering of cells during differentiation (Haghverdi et al. 2015, 2016; Angerer et al. 2016). Briefly, DPT for each cell was derived from its distance to a user-selected root cell, representing the earliest differentiation stage, within a diffusion map (DM) embedding of the data. To create the DM, we applied destiny::DiffusionMap to the 10 PCs of the 2000 most variable genes computed previously. The resulting DM served as the input for destiny::DPT to infer DPT. We selected the root cell as the one with the highest *CD34* expression, a marker for hematopoietic stem cells.

### Differential expression analysis on macrophages within lung tumor

Macrophages from SCLC and LUAD samples were grouped together for comparison with normal macrophages, as differences between cancer subtypes were not of interest. The DE analysis was performed using the Seurat function Seurat::FindMarkers, selecting MAST as the DE method and retaining DEGs that were expressed in at least 10 percent of the tumor macrophages (Finak et al. 2015). The top 50 DEGs of the tumor macrophages were then selected according to their pi-values (Xiao et al. 2014).

## Code availability

All code required to reproduce the analyses and the NeighbourNet R package are available at GitHub (https://github.com/meiosis97/NeighbourNet) and as Supplemental Code.

## Competing interest statement

The authors declare no competing interests.

## Supplemental Material

Supplement 1

Supplement 2
