# Supplementary figures and images for: Scalable cell-specific coexpression networks for granular regulatory pattern discovery with NeighbourNet
Source: Genome Res. 2026 Apr;36(4):785–801. doi: 10.1101/gr.281171.125 (PMC13138013; doi:10.1101/gr.281171.125)

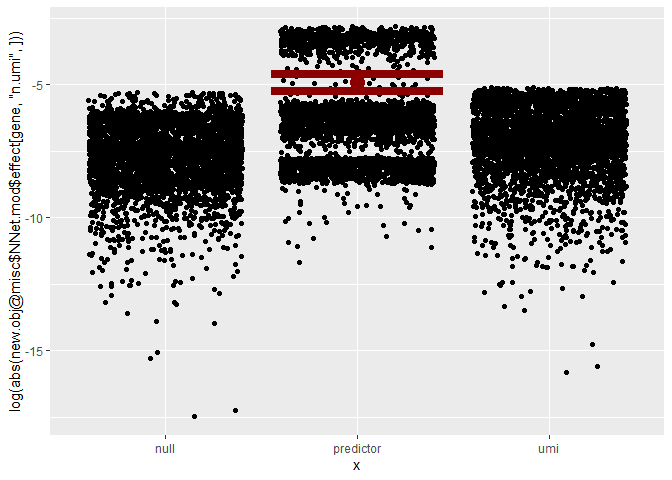

Supplement: Supplement 1 [file Supplemental_Code.zip › code/NeighbourNet-1.01/misc/investigate.pruning_files/figure-markdown_github/unnamed-chunk-10-1.png]

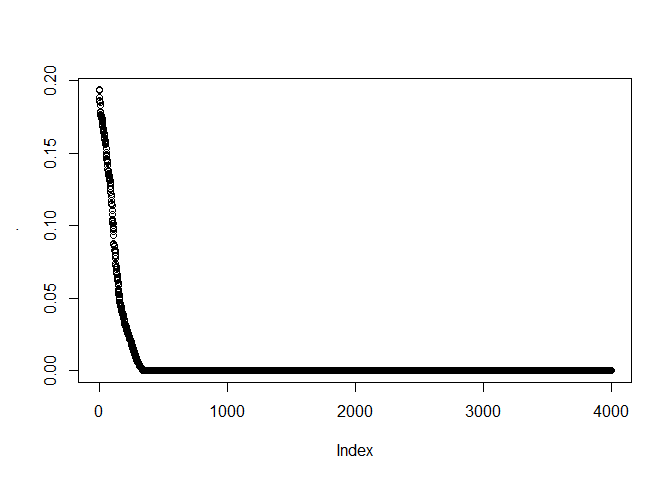

Supplement: Supplement 1 [file Supplemental_Code.zip › code/NeighbourNet-1.01/misc/investigate.pruning_files/figure-markdown_github/unnamed-chunk-11-1.png]

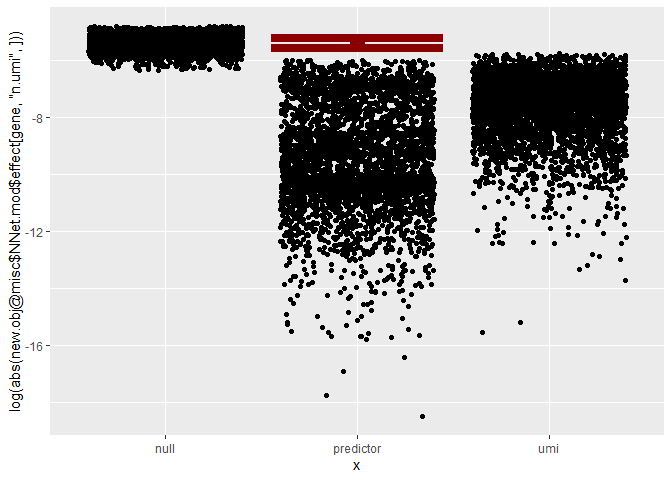

Supplement: Supplement 1 [file Supplemental_Code.zip › code/NeighbourNet-1.01/misc/investigate.pruning_files/figure-markdown_github/unnamed-chunk-12-1.png]

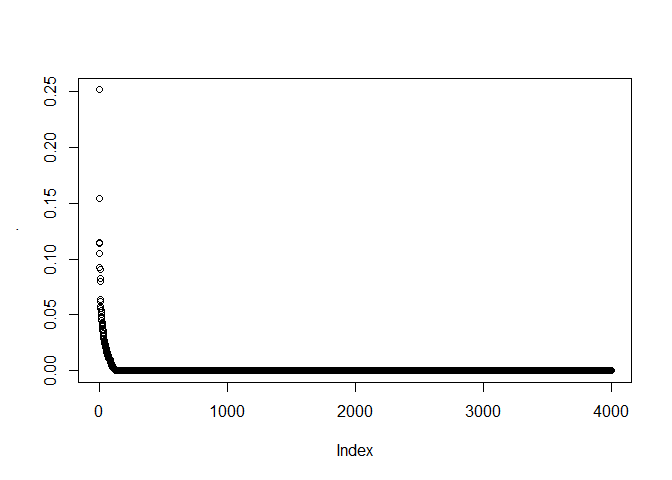

Supplement: Supplement 1 [file Supplemental_Code.zip › code/NeighbourNet-1.01/misc/investigate.pruning_files/figure-markdown_github/unnamed-chunk-13-1.png]

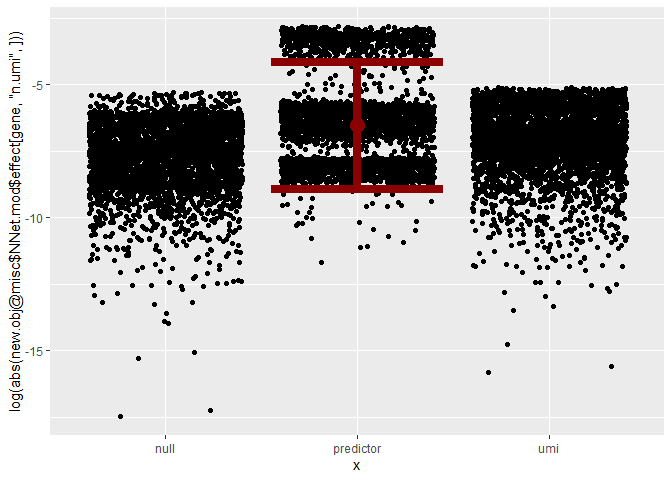

Supplement: Supplement 1 [file Supplemental_Code.zip › code/NeighbourNet-1.01/misc/investigate.pruning_files/figure-markdown_github/unnamed-chunk-6-1.png]

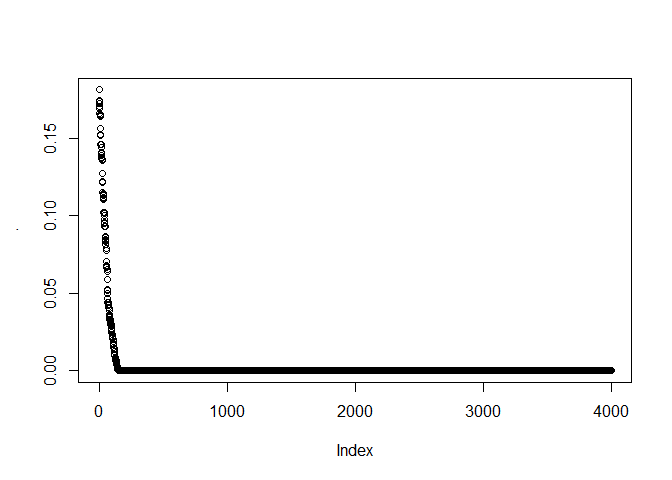

Supplement: Supplement 1 [file Supplemental_Code.zip › code/NeighbourNet-1.01/misc/investigate.pruning_files/figure-markdown_github/unnamed-chunk-7-1.png]

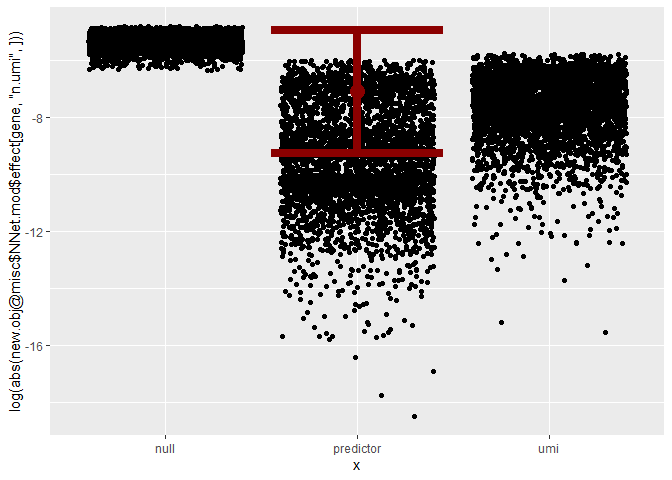

Supplement: Supplement 1 [file Supplemental_Code.zip › code/NeighbourNet-1.01/misc/investigate.pruning_files/figure-markdown_github/unnamed-chunk-8-1.png]

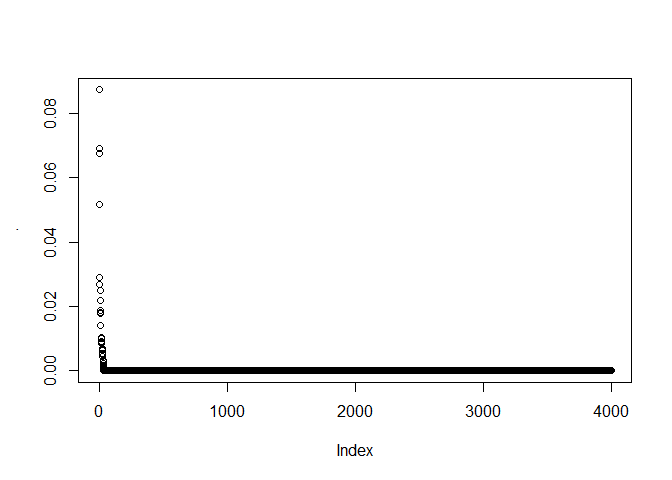

Supplement: Supplement 1 [file Supplemental_Code.zip › code/NeighbourNet-1.01/misc/investigate.pruning_files/figure-markdown_github/unnamed-chunk-9-1.png]

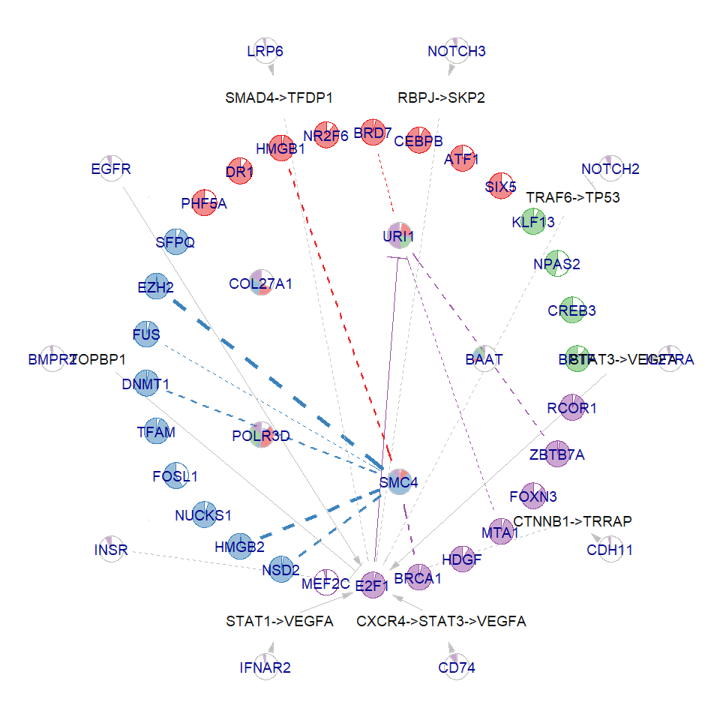

Supplement: Supplement 1 [file Supplemental_Code.zip › code/NeighbourNet-1.01/misc/logo/An_cell_specific_network.png]

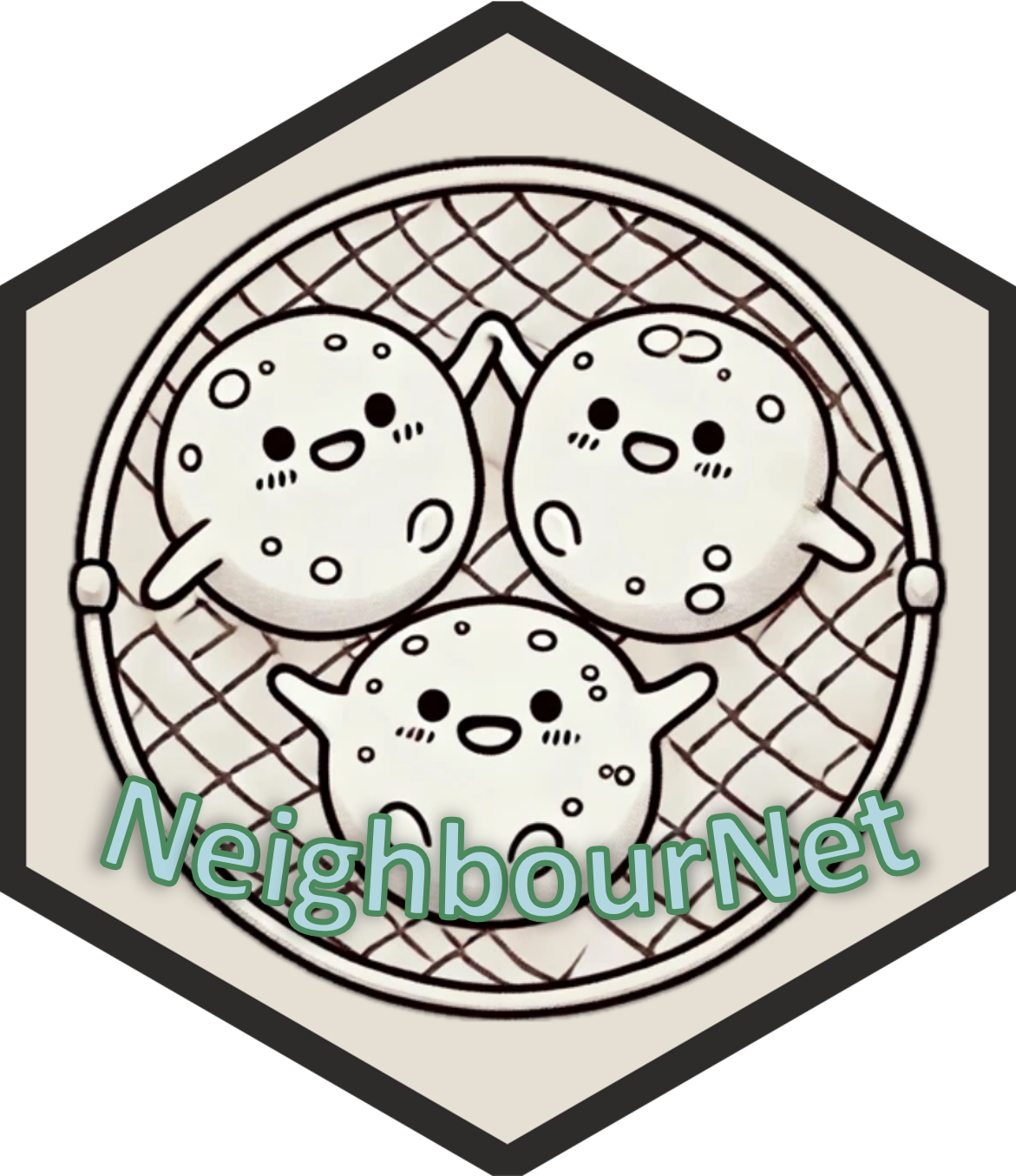

Supplement: Supplement 1 [file Supplemental_Code.zip › code/NeighbourNet-1.01/misc/logo/NNet.png]

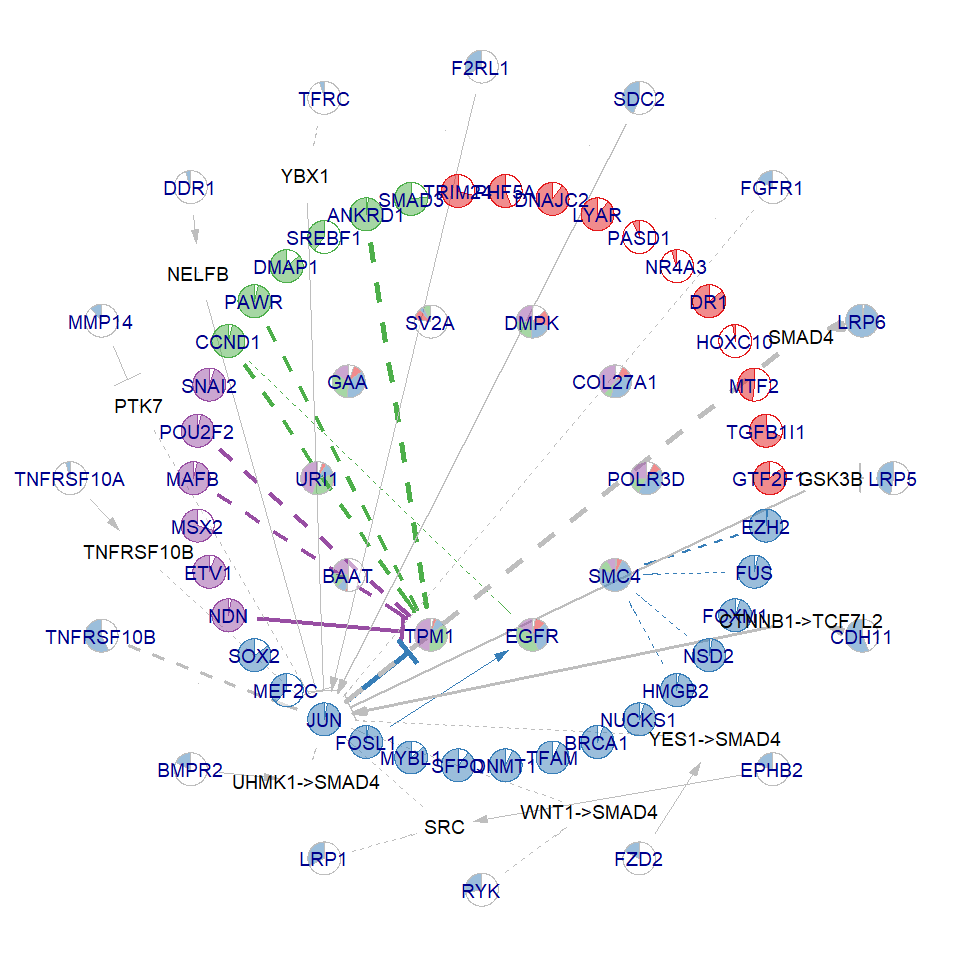

Supplement: Supplement 1 [file Supplemental_Code.zip › code/NeighbourNet-1.01/misc/vignettes.for.script_files/figure-markdown_github/unnamed-chunk-21-1.png]

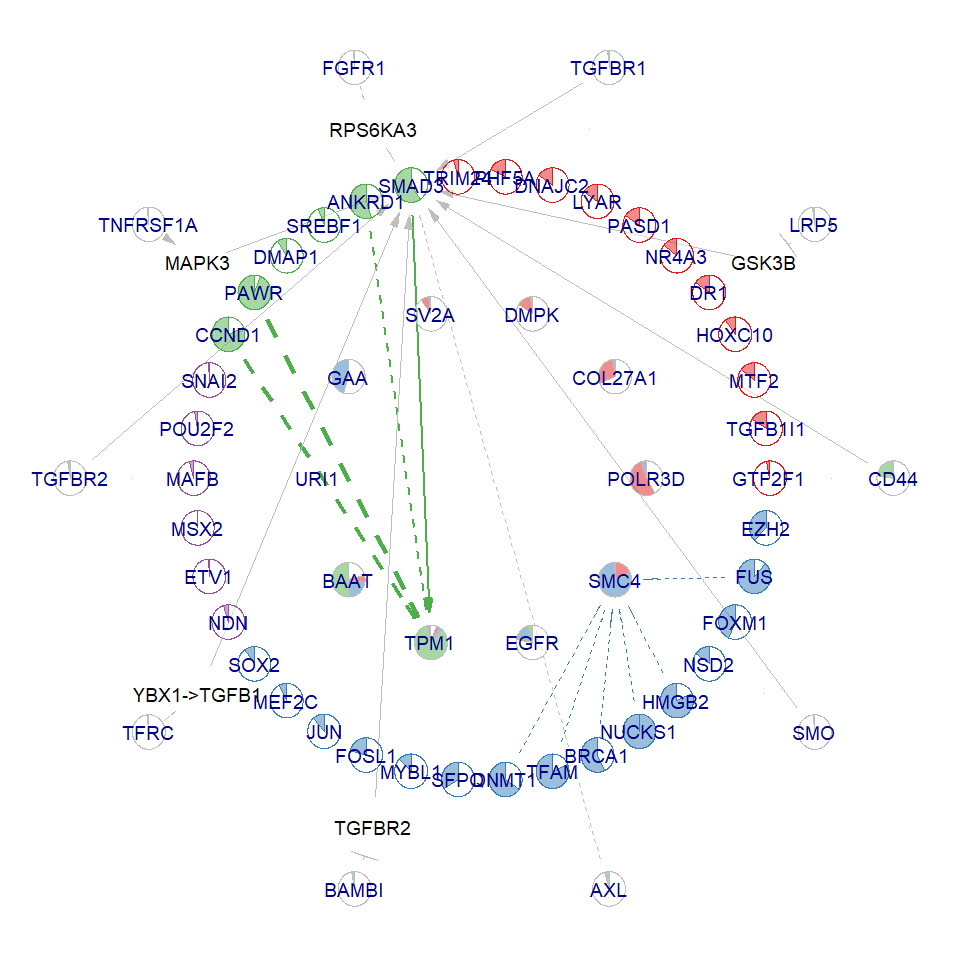

Supplement: Supplement 1 [file Supplemental_Code.zip › code/NeighbourNet-1.01/misc/vignettes.for.script_files/figure-markdown_github/unnamed-chunk-22-1.png]

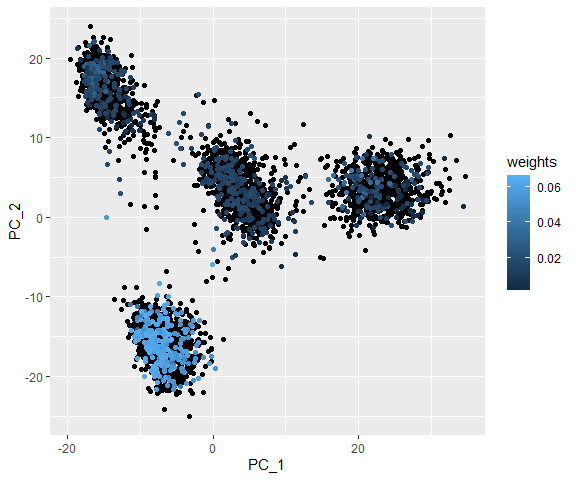

Supplement: Supplement 1 [file Supplemental_Code.zip › code/NeighbourNet-1.01/misc/vignettes.for.script_files/figure-markdown_github/unnamed-chunk-23-1.png]

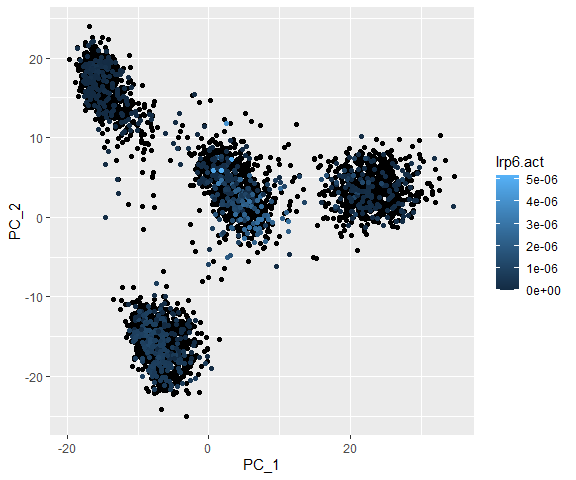

Supplement: Supplement 1 [file Supplemental_Code.zip › code/NeighbourNet-1.01/misc/vignettes.for.script_files/figure-markdown_github/unnamed-chunk-25-1.png]
