# Supplementary material for: Scalable cell-specific coexpression networks for granular regulatory pattern discovery with NeighbourNet
Source: Genome Res. 2026 Apr;36(4):785–801. doi: 10.1101/gr.281171.125 (PMC13138013; doi:10.1101/gr.281171.125)
Supplement: Supplement 2 [file Supplemental_Material.pdf]

Scalable cell-specific coexpression networks for granular regulatory pattern discovery

Yidi Deng[1,2], Jiadong Mao[1,†] & Jarny Choi[3,†] & Kim-Anh Lê Cao[1,*,†]

[1]Melbourne Integrative Genomics, School of Mathematics and Statistics, The University of Melbourne, Australia

[2] Research School of Finance, Actuarial Studies & Statistics, The Australian National University, Australia

[3]Bioinformatics and Cellular Genomics, St Vincent's Institute, Australia

† indicates equal contribution

∗ corresponding author: kimanh.lecao@unimelb.edu.au

S1 Supplementary Results

S1.1 Evaluate linear approximation of permutation feature importance as the coexpression measure

NNet's gene coexpression measure is derived from permutation feature importance (PFI). In the standard procedure, after fitting a PC regression model for a response gene within a cell's neighbourhood, we permute the expression values of a predictor gene within the neighbourhood while keeping all other predictor genes unchanged. The permuted data is then projected onto the global PCA loading, and the resulting PCs are fed into the fitted regression model to predict the response. PFI for the permuted predictor gene is calculated as the expected squared difference between the prediction made before and after permutation. However, directly permuting each predictor gene and making repeated predictions for thousands of cell neighbourhoods is computationally prohibitive.

To address this, we derived a linear approximation of PFI that can be easily calculated, bypasses the permutation-and-prediction procedure (Supplementary Methods S3.3.2). Here, we evaluate how well this approximation performs compared to the PFI scores calculated using actual permutation.

We utilised a human lung adenocarcinoma cell line (HCC827, H1975, A549, H838 and H2228) dataset, containing 3,918 cells, for evaluating the goodness of the approximation (Tian et al., 2019). We downloaded the processed data from https://github.com/LuyiTian/sc_mixology/tree/master/data (Last retrieved: Dec 12th, 2024). PCA was conducted on the 2,000 most variable features (VFs) to generate global PCs. We then randomly selected cells and their neighbouring cells to perform PC regression using different regression methods: ridge regression, random forest (RF), and support vector machine (SVM) with a radial kernel. For each randomly sampled cell, a gene from the 2,000 VFs was randomly chosen as the response, and another gene was selected to measure its PFI and approximated PFI in predicting the response. This procedure was repeated 1,000 times for different cells and genes, with PFI scores calculated using both actual permutation and the approximation for each regression method. We then evaluated the correlation between the approximated and actual PFI scores across the iterations.

The results demonstrate that the approximated PFI aligns perfectly with the actual PFI for linear regression methods such as ridge regression, showing near-perfect correlations. For SVM with a radial kernel, the approximation also performed exceptionally well, with correlations exceeding 0.9, indicating strong concordance between the approximated and actual PFI. However, the approximation was less effective for RF, yielding weaker correlations. This discrepancy may be due to the smaller sample size within each neighbourhood, which exacerbates the discontinuous nature of RF predictions. Since the PFI approximation relies on estimating partial derivatives, discontinuities in RF predictions likely introduce errors, reducing the accuracy of the approximated PFI.

Overall, our linear approximation of PFI provides a highly efficient and accurate alternative to permutation-based PFI for linear models and SVMs, significantly reducing computational burden while maintaining reliability. While it is less effective for RF, this limitation underscores the dependency of the approximation's accuracy on the smoothness of the underlying regression model's

predictions.

S1.2 Sensitivity and specificity analysis of NNet coexpression pruning based on significance measures

We performed a sensitivity (false positives rate) and specificity (true negative rate) analysis to evaluate the effectiveness of NNet pruning in distinguishing meaningful from irrelevant gene associations using the proposed significance measure on coexpression (Supplementary Methods S3.3.5). A key feature of PC regression, leveraged in this analysis, is its ability to measure coexpression between response genes and themselves (self-coexpression) when these response genes are also used to embed PCs. Since a gene should inherently exhibit a significant self-coexpression, this provides a natural benchmark for assessing the pruning process. Specifically, we examined whether NNet pruning successfully retains these self-coexpression, which represent true positive associations, while filtering out irrelevant or non-significant coexpression, minimising false discoveries.

We used the human lung adenocarcinoma cell line dataset described in Section S1.1 for this analysis, focusing again on the 2,000 most VFs. To evaluate specificity, we first extracted the expression data of these VFs and generated a permuted dataset as a negative control, where each VF was permuted independently. The original and the permuted dataset were concatenated, forming a single dataset with 4,000 features (2,000 original VFs and 2,000 permuted VFs). PCs were computed on this concatenated dataset, and NNet analysis was performed using each un-permuted VF as the response. This allowed us to measure and compare each VF's self-coexpression and the coexpression with its permuted counterpart across cells. A pruning selection was considered a true positive if a VF's self-coexpression was identified as significant in a cell, and a false positive if the coexpression with the VF's permuted counterpart was identified as significant. Using this framework, we calculated the sensitivity and specificity for each VFs.

For each VF, we analysed cells in two strata: those expressing the VF and those not expressing it. This is because coexpression can only be detected when the gene is expressed. Results are summarised in Supplementary Figure S6A.

- **Default (heuristic) pruning** In VF-expressing cells, NNet's default settings yielded an

average F1-score of ≈ 0.77 for identifying self-co-expression. As expected, sensitivity was lower in cells that did not express the VF. Specificity was near-perfect in both strata: co-expression with permuted counterparts was virtually always rejected, confirming that the default settings are conservative and favour low false-positive rates.

- **Alternative housekeeping-gene pruning** (Supplementary Methods S3.3.5). This more rigorous strategy raised the mean F1-score for VF-expressing cells to ≈ 0.79. However, performance was inconsistent: a subset of VFs showed F1-scores ¡ 0.25. Thus, although the alternative approach can improve accuracy on average, its instability across targets makes it less reliable for routine analyses.

Given this trade-off, we adopted the default pruning strategy in the main study. However, it is worth noting that the default strategy generates a null distribution of coexpression based on permuting self-coexpression over the KNN graph smoothing. Therefore, its null distribution is essentially the bootstrap distribution of the averaged self-coexpression. Consequently, the benchmark for power is intrinsically tilted in favour of the default procedure.

To further assess the impact of pruning, we inspected VFs for which either strategy showed very low sensitivity or specificity. We compared the distributions of importance scores for each VF's self-co-expression and co-expression with its permuted counterparts against the null importance distributions for pruning described in Supplementary Methods S3.3.5. The alternative strategy generates broader nulls than the default, explaining its higher pruning instability. By contrast, the default strategy produces much narrower nulls. As a result, changing the significance threshold rarely changes the pruning outcome, limiting the scope for fine-tuning sparsity. Both strategies performed poorly when a VF was expressed ubiquitously, and every instance of self-co-expression should in principle be retained. The default strategy, nevertheless, enforces a split between significant and non-significant co-expression, as illustrated in Supplementary Figure S6C, whereas the alternative benchmark can yield nulls that are misaligned with the distribution of observed self-coexpression (Supplementary Figure S6B). On balance, we therefore prioritised the default approach, accepting a modest loss of power in exchange for the greater robustness and reproducibility it provides.

S1.3 Evaluation of tuning parameters

We evaluated the sensitivity of NNet results to changes in key tuning parameters, including: (i) the number of PCs used in regression ($nPCs$), (ii) the neighbourhood size (KNN) used to define local regression, and (iii) the number of steps t in the diffusion random walk used to smooth coexpression along the KNN graph. Analyses were performed on the PBMC 3K dataset. Following the preprocessing described in Material & Methods, we extracted 731 TFs, which were used as responses to construct TF–TF cell-specific coexpression networks (CSNs) across 2,638 cells.

We conducted two evaluations of the resulting CSNs:

1. **Self-coexpression ranking.** Since the TFs used as responses are also embedded within the PCs, we calculated each TF's self-coexpression within each CSN. As self-coexpression should be higher than coexpression with other TFs, we ranked each TF's self-coexpression relative to all its coexpression values and averaged these ranks across cells. Each tuning setting thus produced 731 averaged self-ranks, which were compared across parameter settings.

2. **Clustering by TF degree.** For each CSN, we calculated TF degrees and performed clustering of cells based on their TF degree profiles. Clustering quality was quantified using the median silhouette index (MSI) of cell-type clusters, computed from the distance between degree profiles.

We evaluated nine different parameter settings, grouped by which parameter was varied while the others were fixed at their defaults ($nPCs = 57$, chosen automatically by NNet via the spectral rule; $KNN = 30$; $t = 3$):

1. **Varying $nPCs$:** 30, 50, and 100 PCs.

2. **Varying KNN:** 15, 50, and 100 neighbours.

3. **Varying t:** $t = 1$, $t = 5$, and $t = 10$.

Results are illustrated in Supplementary Figure S5. Self-ranking scores were consistently high (above the 95th percentile; Supplementary Figure S5A), indicating that NNet reliably captures

genuine coexpression. Parameter effects were observed: increasing $nPCs$ improved self-ranking scores. This is likely because more predictors add information; in the limit where PCs equal the number of predictors, perfect self-coexpression is expected. However, differences between 50 and 100 PCs were minimal, supporting the effectiveness of the default choice (57 PCs). Smaller KNN and larger t place greater emphasis on local coexpression. Consistent trends were observed: as local emphasis increased, self-ranking scores became higher. This suggests that local coexpression is more likely to capture genuine regulatory signals than coexpression estimated globally across mixed cell populations.

Clustering performance, as measured by MSI (Supplementary Figure S5B), was largely unaffected by $nPCs$ and KNN. In contrast, t had a stronger effect: larger values restricted local variation and yielded higher MSI, as expected.

In conclusion, NNet is robust to tuning parameters, and the default settings provide a well-balanced choice.

S1.4 Benchmarking existing cell-specific methods

Due to the difficulty of simulating CSNs, we performed a minimal benchmark to test whether NNet-inferred CSNs preserve basic biological information: namely, whether variation in network structure reflects cell-type differences. We focus on node degree of CSNs, calculate cell-specific degree profiles and assesse whether these profiles preserves known cell-types.

For this evaluation, we used the PBMC3K dataset, which was preprocessed and annotated as described in Material & Methods. We compared NNet with two existing cell-specific methods:

1. **oCSN** (Dai et al., 2019), the first paper to introduce the concept of CSNs, which measures cell-specific coexpression as the correlation between two genes within a cell's neighbourhood, defined by a fixed window around the two genes' expression.

2. **LocCSN** (Wang et al., 2021), an extension of oCSN that instead defines neighbouring cells with an adaptive window.

Using all three methods with default tuning parameters, we inferred TF–TF CSNs (731 TFs)

and constructed degree matrices reflecting TF degrees across cells. To assess whether inferred TF degree preserved cell-type identity, we computed the first 10 PCs of the degree matrix and then calculated the median silhouette index (MSI) of the known cell-type labels on these PCs. For fairness, we did not apply network smoothing in NNet, as smoothing depends on the KNN graph and could artificially enforce clustering, potentially giving NNet an advantage.

Results are shown in Supplementary Figure S7A. NNet preserves cell-type structure more clearly, whereas the two pioneering methods (oCSN and LocCSN) performed poorly in this respect. Regarding scalability, oCSN (implemented in MATLAB) is extremely fast, while NNet also produces results within a reasonable runtime. Importantly, for NNet, runtime does not scale with the number of targets: constructing TF–target CSNs (731 $\times$ 4,400) requires the same time as TF–TF CSNs (731 $\times$ 731), underscoring NNet's scalability advantage for larger networks.

Supplementary Figure S7B further illustrates the NNet workflow. NNet integrates seamlessly with Seurat and can perform CSN inference, meta-network analysis, and signalling inference with just a few lines of code. By contrast, neither oCSN, LocCSN, nor any other existing cell-specific methods provide this level of accessibility for downstream analysis of inferred CSNs.

S1.5 Extra Perturb-seq analysis

We performed an additional evaluation of expression-based (ETAS) and coexpression–based (Co-ETAS) scores on a separate Perturb-seq dataset, independent of the one analysed in Case study 1: TF activity inference.

The data used was originally generated by Dixit et al. (2016), who established the scRNA-seq protocol for pooled CRISPR perturbation on multiple loci of individual cells to discern genetic interactions. We downloaded the cleaned version of the data produced from the repository of Holland et al. (2020) (`https://zenodo.org/records/3564179`), who conducted a systematic benchmark of TF activity inference methods using this data. The data comprises 10,000 genes and includes a total of 26,139 cells, each of which could be perturbed with a random combination of 10 different TFs (*EGR1*, *NR2C2*, *E2F4*, *CREB1*, *YY1*, *GABPA*, *IRF1*, *ELK1*, *ELF1*, *ETS1*), or perturbed inter-genetically as negative controls. The cells were sequenced at two time points: 16,506 cells

at 7 days and 9,633 cells at 13 days post-perturbation. We did not perform additional quality control to filter out genes or cells. The data was then log-normalised using the Seurat function `Seurat::NormalizeData` with its default tuning.

The NNet analysis was performed on all the cells using the 10 perturbed TFs as responses. We applied the same procedure as in the PBMC data (Material & Methods) to select 3,227 targets in CollecTRI as predictors. decoupleR activity scores were calculated for each of the 10 perturbed TFs in both perturbed and unperturbed cells. The area under the curve (AUC) was used to evaluate the effectiveness of these scores in distinguishing perturbed (negative class) from unperturbed cells (positive class). The perturbation effect increased with time, as evidenced by higher AUC values for both ETAS and CoETAS (Supplementary Figure S1D1). While CoETAS performed comparably to ETAS on day 7, its superior performance on day 13 suggests that NNet coexpression offers a more accurate detection of TF activation status.

S2 Supplementary Figures

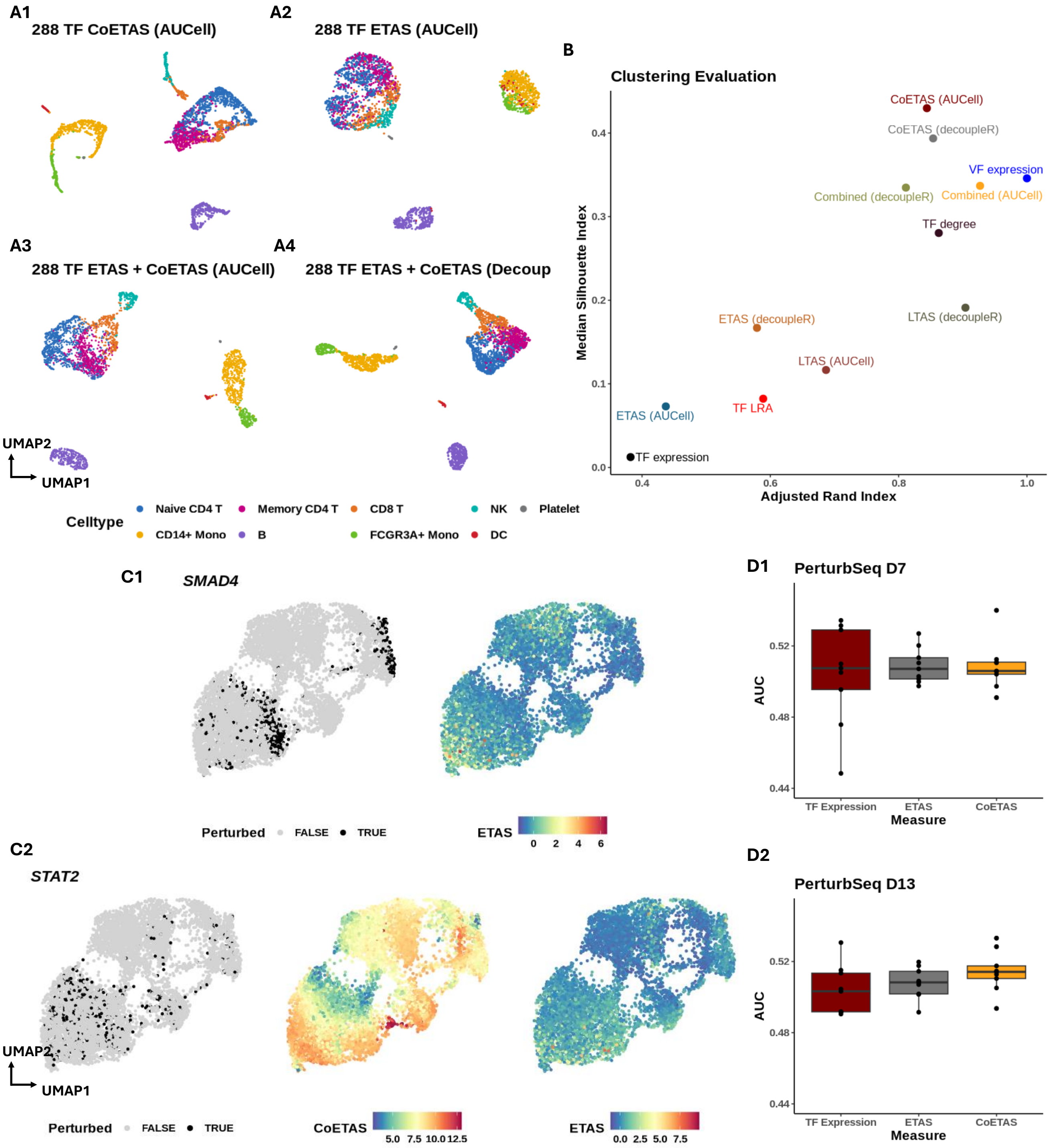

Figure S1. TF activity case study (Case study 1: TF activity inference). **(A)** Similar to Figure 2B, UMAP visualisation of the 10x Genomics PBMC3K dataset, embedded using transcription factor (TF) activity scores inferred by AUCell (A1, A2). We further evaluated whether combining expression-based (ETAS) and coexpression-based (CoETAS) scores can improve clustering quality. Panels (A3) and (A4) show UMAP embeddings based on the concatenated CoETAS + ETAS matrices, with TF activity scores inferred using AUCell (A3) or decoupleR (A4). **(B)** An extension of Figure 2C, comparing clustering schemes of the PBMC3K data using 10 principal components (PCs) derived from various TF activity measures. The additional measures include: cell-specific TF degree; concatenated TF activity scores from CoETAS and ETAS inferred by AUCell (Combined-AUCell) or decoupleR (Combined-decoupleR); and TF activity based on the low-rank approximation (LRA) of gene expression inferred by AUCell (LRA-AUCell) or decoupleR (LRA-decoupleR). The LRA of 288 TF expressions (TF-LRA) is included as a control. **(C)** *SMAD4* (C1) and *STAT2* (C2) perturbation status and activity inferred on Papalexi et al. (2021). **(D)** Benchmarking on the Perturb-seq dataset from Dixit et al. (2016): For 10 TF knockdowns, ETAS and CoETAS were computed using decoupleR and evaluated by their ability to distinguish perturbed cells from controls using the area under the curve (AUC). TF expression served as a baseline. Results at days 7 (D1) and 13 (D2) show that CoETAS outperformed ETAS, particularly at day 13.

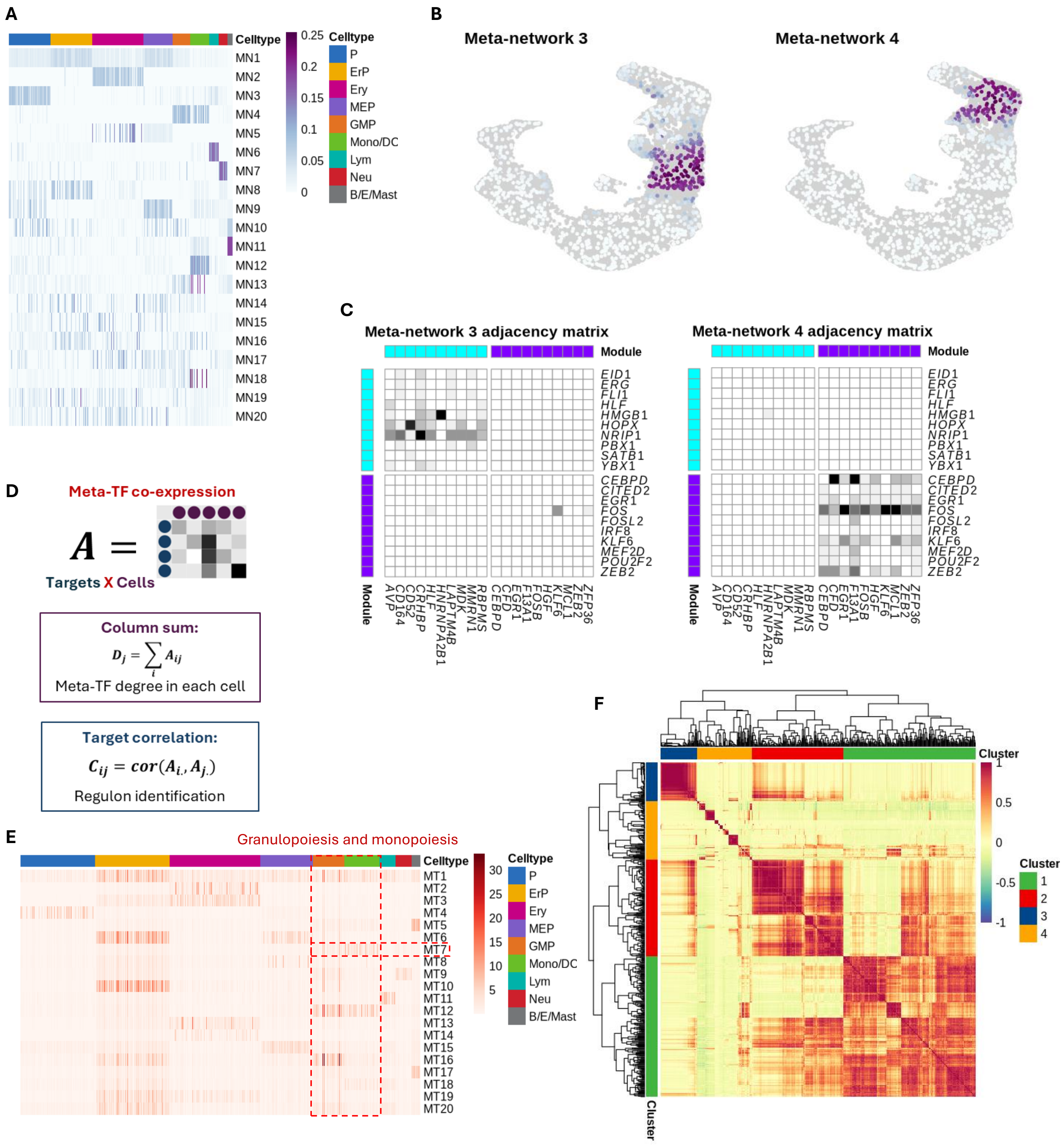

$$A = $$

$$D_j = \sum_i A_{ij}$$

$$C_{ij} = cor(A_{i,}, A_{j,})$$

Figure S2. Meta-network case study on early hematopoiesis (Case study 2: The early hematopoiesis study). **(A)** Heatmap of cell weights derived from NMF, which were used to embed the cell dimension of the network ensemble. Each column represents one of the 20 weighting vectors used to construct the 20 meta-networks. The weight of a cell indicates its relevance to the corresponding meta-network. **(B)** Cell weights on meta-network 3/4. **(C)** Weighted adjacency matrix representation of meta-network 3/4. The top ten most connected TFs and their targets were selected within each of the meta-networks, represented as rows and columns, respectively. Genes are grouped by the meta-network from which they were extracted. **(D)** Individual TF coexpression with targets across cells (represented as a target-by-cell matrix) can be aggregated the soft-clusters of TFs. Each cluster represents a TF module that contributes to constructing a meta-TF, with the weights indicating the contribution of individual TFs. Aggregation yields cell-specific Meta-TF-target coexpression networks. These network works can be analysed by (i) summing target coexpression within each cell to quantify meta-TF activity, and (ii) calculating correlations between targets to identify the meta-TF regulon. **(E)** Similar to (A), but the rows of this heatmap illustrate the top 20 meta-TFs' node degree across cells. Meta-TF 7 was differentially connected in the mononuclear-phagocyte lineage according to our first analysis proposed. **(F)** Target correlation calculated based on their coexpression with meta-TF 7. From hierarchical clustering we identified four major regulons.

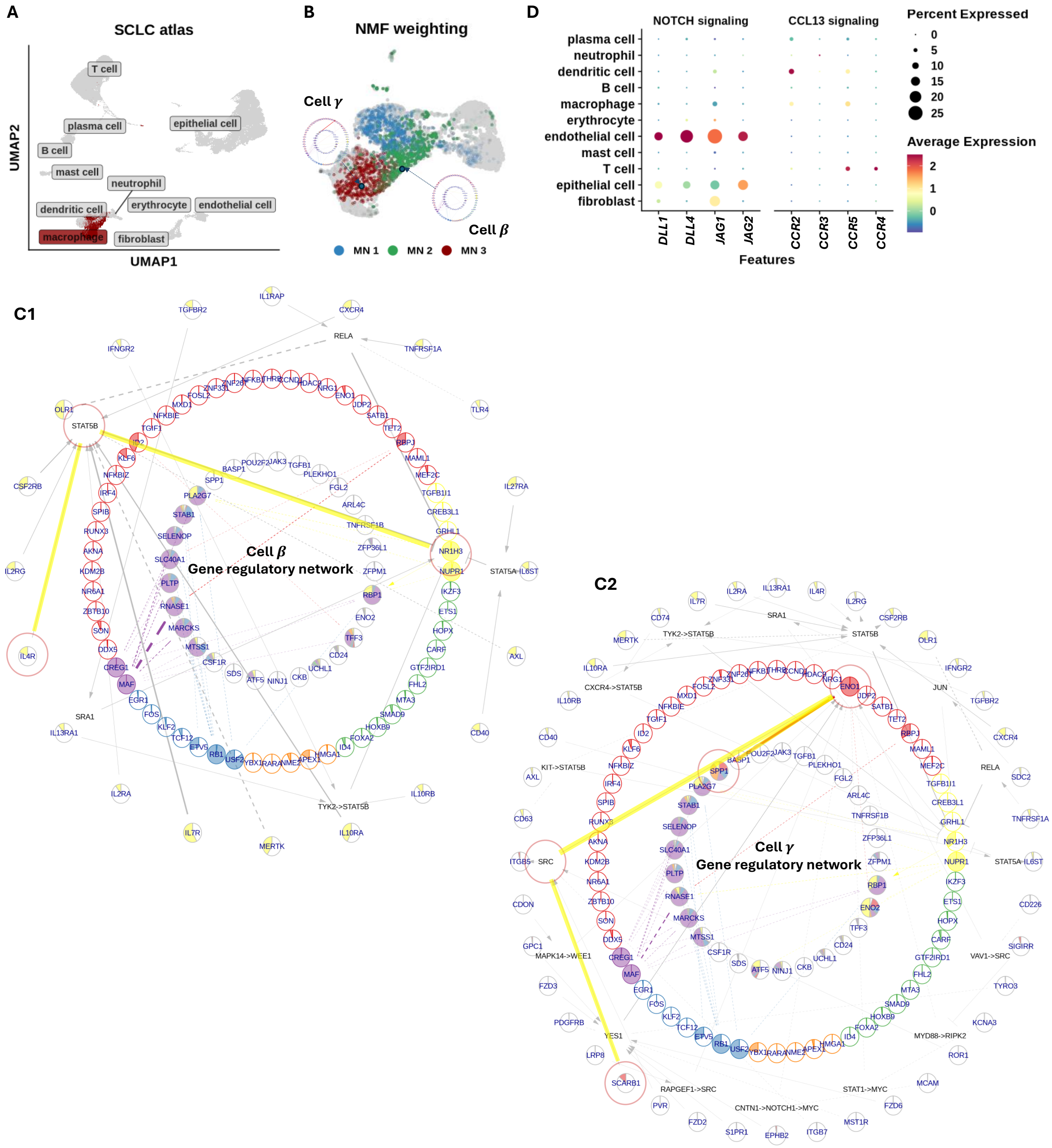

Figure S3. Prior knowledge annotation case study on tumour associated macrophage (Case study 3: The lung cancer study). (A) UMAP visualisation of the small cell lung cancer scRNA-seq data of Chan et al. (2021). The UMAP embedding was pre-computed and stored by the CELLxGENE scRNA-seq data portal. **(B)** Continue of panel (D) from Figure 4. Cell β and cell γ, which have the highest cell weights on meta-network 2 and 3, are highlighted respectively. **(C)** Prior knowledge annotation of the coexpression network of cell β (C1) and cell γ (C2). Cell γ displays a dual identity involved in a NR1H3-mediated pro-inflammatory response, undergoing suppression directed by anti-inflammatory signals from *IL4R* and *IL13R*. Cell γ exhibits a decreased level of *NR1H3* connection and an establishment of a connection between *ENO1* and *SPP1*, characteristic of an SPP1+ pro-tumourigenic macrophage. According to our upstream signalling pathway (USP) inference, a top USP candidate for *ENO1*-mediated *SPP1* signalling in cell γ is *SCARB1*, with its signal likely transduced via SRC. **(D)** The dot plot illustrates the expression levels of key ligands involved in the NOTCH signalling pathway alongside the expression of the *CCL13* receptor across various cell populations. Endothelial cells could be the major source of NOTCH signalling that contributes to tumour associated macrophage development. As a response, macrophages of intermediate states release *CCL13* that predominantly targets immune cells.

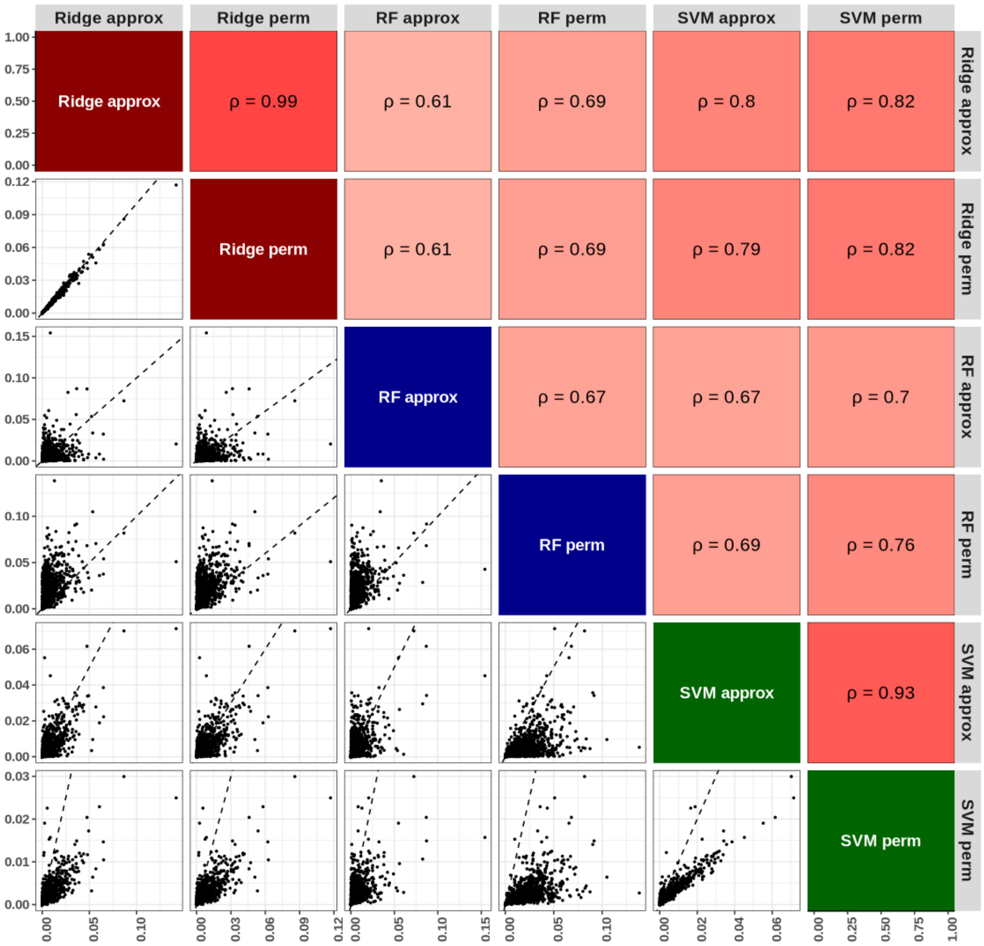

Figure S4. Evaluate linear approximation of permutation feature importance as the coexpression measure (Supplementary Results S1.1). Biplot assessing the concordance between permutation feature importance (PFI) scores derived using the linear approximation described in Supplementary Methods S3.3.2 and those calculated via actual permutation. Lower triangle: scatter plots of PFI scores computed with different regression models (Ridge: ridge regression, RF: random forest, SVM: support vector machine), comparing the linear approximation and actual permutation. Dashed lines represent identity lines. Upper triangle: correlation coefficients between PFI scores derived from different approaches. The figure demonstrates high correlation between approximated and actual PFI scores for linear models and SVMs, showcasing the accuracy and computational efficiency of the approximation

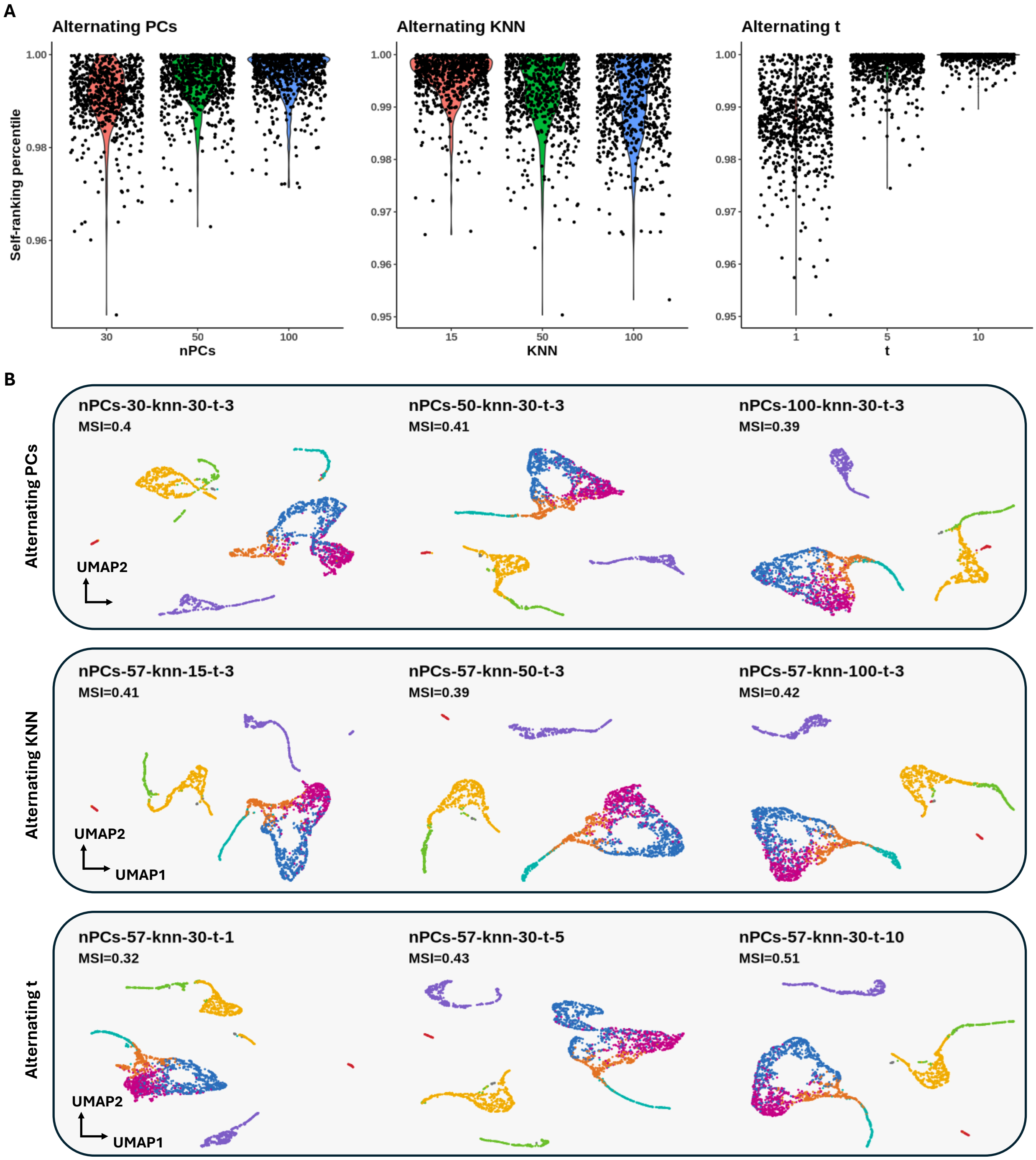

Figure S5. Effect of tuning parameters on NNet performance on the PBMC3K data (Supplementary Results S1.3). Evaluation of tuning parameters in NNet. We constructed TF–TF cell-specific coexpression networks (CSNs) for 2,638 cells from the PBMC3K dataset, using 731 TFs as responses, and assessed how network properties change under different parameter settings: the number of PCs ($nPCs$), neighbourhood size (K), and diffusion steps (t). Nine tuning settings were tested, each varying one parameter from the default ($nPCs$ = 57, KNN = 30, t = 3). **(A)** Self-coexpression ranking. For each TF, self-coexpression within each CSN was ranked relative to its coexpression with other TFs, and averaged across cells. Each point represents the averaged self-ranking of a TF in a specific tuning setting. Rankings remain consistently high ($> 95^{th}$ percentile) across all settings, confirming that NNet robustly captures genuine coexpression. Smaller KNN and larger t emphasise local estimation, yielding higher self-ranking scores. (B) Clustering performance. Cells were clustered based on TF degree profiles, and performance was quantified by the median silhouette index (MSI). While $nPCs$ and KNN had little effect, larger t values increased MSI by restricting local variation, potentially suggesting over-smoothing.

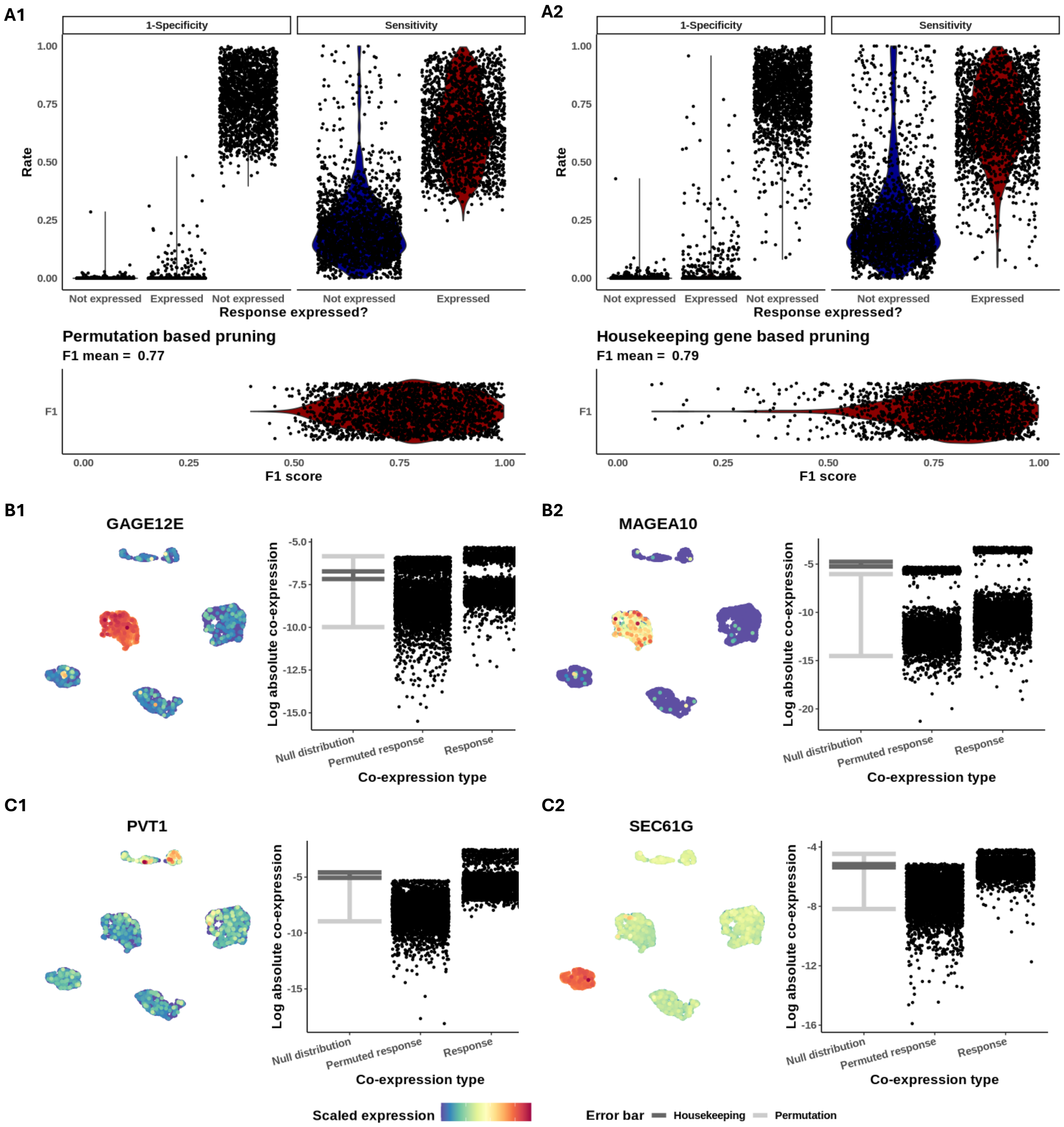

Figure S6. Sensitivity and specificity analysis of NNet pruning based on significance measures (Supplementary Results S1.2). (A) A specialised dataset was constructed for this analysis, comprising the 2,000 most variable features (VFs) from the human lung adenocarcinoma cell line dataset of Tian et al. (2019), along with a permuted counterpart for each VF. Using NNet, we evaluated each VF as a response gene and assessed the pruning effectiveness in three aspects: sensitivity (the ability to retain a response gene's coexpression with itself as significant), specificity (the ability to filter out the response gene's coexpression with its permuted counterpart), and F1 score that combines the previous two matrices. The analysis was conducted separately on cell populations expressing and not expressing the response gene, using the default heuristic pruning strategy implemented in the main (A1), as well as using the alternative statistical rigorous strategy described in Supplementary Methods S3.3.5 . F1 scores are only evaluated on the 'express' population. Results are visualised as violin plots, with each jittered scatter point representing the outcome for a specific response gene.**(B)** Examples showcasing the response gene, on which NNet default (B1) and alternative (B2) pruning obtained the lowest specificity. The left of each panel demonstrates the gene expression on UMAP; the right is the scatter plot of cells that compares responses' self-coexpression and their coexpression with permuted counterparts, against the null distribution of coexpression generated by the default (dark grey) and the alternative (light grey). **(C)** Similar to (B), but showing genes on which the lowest pruning sensitivities were obtained.

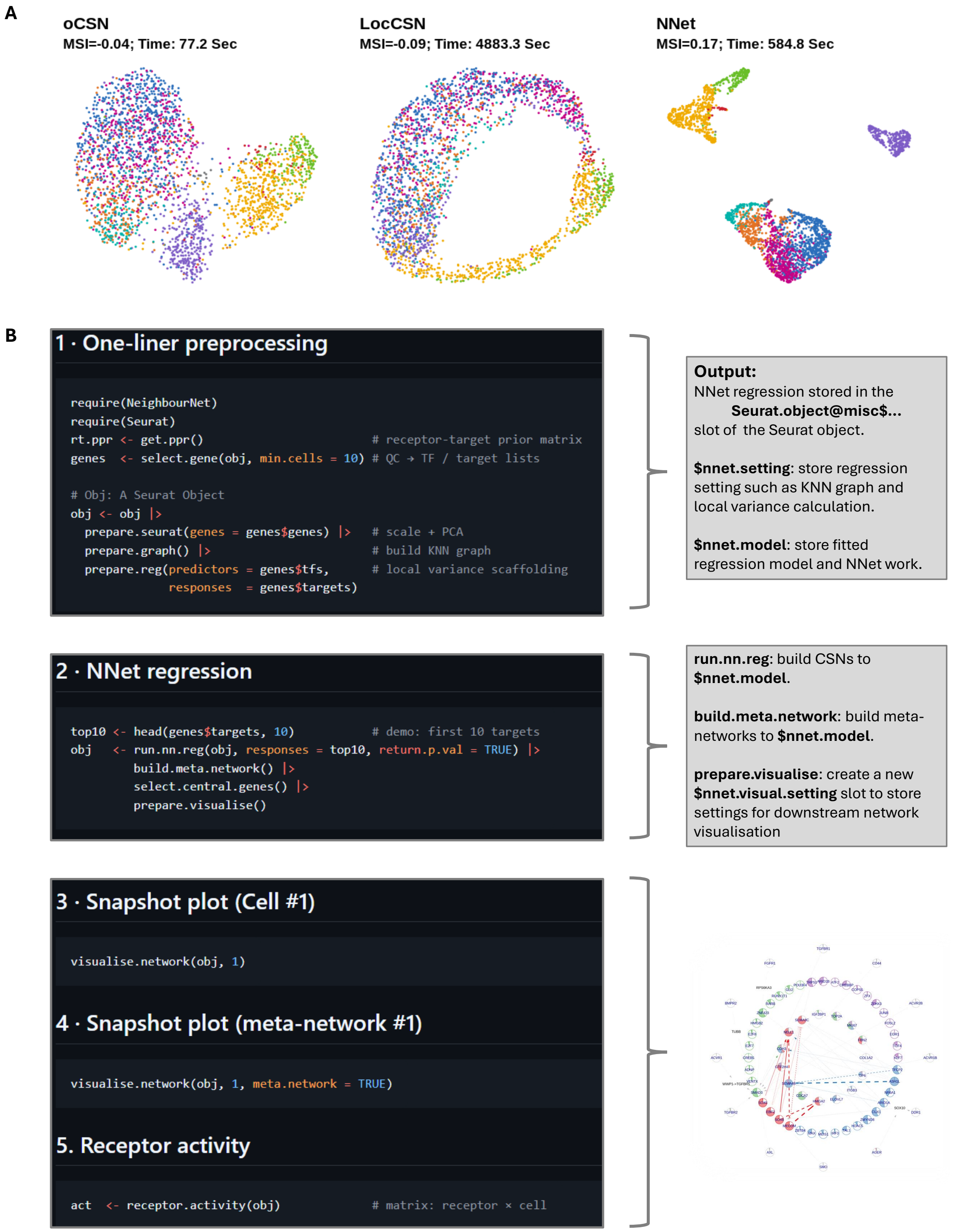

Figure S7. Benchmarking existing cell-specific methods. (Supplementary Results S1.4). We benchmarked NNet against two other cell-specific methods, oCSN (Dai et al., 2019) and LocCSN (Wang et al., 2021), using the PBMC3K dataset. All methods were used to construct TF–TF cell-specific networks (CSNs) containing 731 TFs. For each method, cell-specific TF degrees were computed, and the resulting degree matrices were evaluated based on their ability to preserve first-level cell type information. **(A)** UMAP visualisation of the degree matrices, with cell type preservation performance quantified by the median silhouette index (MSI) and running times reported in seconds. NNet distinguishes cell types more clearly than both methods, with reasonable runtime performance (second only to oCSN). Notably, for NNet, constructing TF–target CSNs (731×4400) takes the same runtime to constructing TF–TF CSNs (731×731), highlighting its scalability advantage when building larger networks. **(B)** NNet workflow. NNet integrates seamlessly with state-of-the-art single-cell analysis pipelines: Seurat. Its modular functions enable CSN inference, meta-network analysis, and upstream signalling inference to be performed within just a few lines of code.

S3 Supplementary Methods

S3.1 Abbreviations

- scRNA-seq: single cell RNA-sequencing
- CSN: cell-specific coexpression network
- TF: transcriptional factor
- PC: principal component
- KNN: k-nearest neighbours
- PCA: principal component analysis
- nPCA: non-negative principal component analysis
- UMAP: uniform manifold approximation and projection
- PPR: personalised page-rank
- LRA: low-rank approximation
- NTF: non-negative tensor factorisation
- NMF: non-negative matrix factorisation
- SVD: singular value decomposition
- PKN: prior knowledge network
- USP: upstream signalling pathway
- PLS: partial least square regression

S3.2 An overview

NNet builds a gene coexpression (or regulatory) network for each cell based on single-cell RNA-seq (scRNA-seq) data. The methods consists of two major components:

1. Building cell-specific networks (CSNs; Section S3.3). This component infers gene coexpression using principal component (PC) regression within each cell's k-nearest neighbours (KNNs).

2. Downstream analysis. This includes meta-network construction (Section S3.4) and function annotation (Section S3.5) of the inferred coexpression networks using prior knowledge in gene regulation and signalling interactions.

Additionally, Section S3.6 describes a subsampling method to address the memory usage challenges associated with loading and analysing large networks of thousands of cells.

We briefly describe the PC regression and prior network annotation below:

S3.2.1 Cell-specific KNN-PC regression

A detailed description of the method can be found in Section S3.3. Here we present an abstract of the method in a sequential order. Steps that are performed globally on the full data and locally on each cell's KNN are noted as *Global* and *Local* respectively.

1. (Global) Principal component analysis (PCA) on the full scRNA-seq data (Section S3.3.1).

2. (Global) Construct a cell k-nearest neighbour graph based on PCA. The graph defines the neighbouring cells of each cell and their similarity in gene expression. The local coexpression network for each cell is then learned within its defined neighbourhood.

3. **For each gene in a response gene sets, repeat step 4 to 6**

4. (Local) Within each cell's neighbourhood, fit a PC regression model on the expression of the response gene.

5. (Local) On each model fitted, calculate importance of genes in predicting the response based on permutation feature importance. The importance score of a gene serves as a measure of its coexpression with the response at the corresponding cell level (Section S3.3.2).

6. (Global) Apply random walk diffusion to denoise the cell by predictor gene importance score matrix of the response (Section S3.3.4).

On each cell we measure coexpression between multiple response genes and predictor genes, which builds a coexpression network. Each cell has its own network (i.e., CSN), and the collection of networks builds a network ensemble (a cell-by-gene-by-gene data entity storing networks, see Section S3.3.3 for details). An optional downstream analysis is applying non-negative PCA (nPCA) on cell space of the network tensor to extract meta-networks that represent principal coexpression patterns (Section S3.4).

S3.2.2 Prior knowledge annotation of coexpression networks

A detailed description of the method can be found in Section S3.5. We have two major goals in this part of method development. The first is to discovery context-specific activation of gene regulation. Genes that are both known to interact based on prior knowledge and are coexpressed in the study context are more likely to represent on-going gene regulation. The second goal is to identify upstream signalling pathways and receptors that could potentially influence the expression of a target gene, given its coexpression with transcription factors (TF). To achieve these goals, we

1. (Pre-computed) Constructed large prior knowledge networks of gene regulation (TF-targets) and protein-protein interaction integrated from multiple confidential databases (Türei et al., 2021). The integration approach we took was acquiesced from Browaeys et al. (2020). The prior knowledge network is included in the R package.

2. (Pre-computed) Build a receptor-TF regulatory potential matrix by running personalised page-rank algorithm (PPR) on the prior knowledge network.

3. (Local) Quantify receptors' regulatory activity on targets by integrating prior knowledge in receptor-TF regulatory potential with target-TF coexpression. Receptors that strongly correlates with a target through TFs are more likely to be the signalling transductor of target gene expression.

S3.3 Cell-specific KNN-PC regression

We infer gene coexpression using regression models, which quantify the relationships between genes by estimating the importance of predictor gene expression in predicting response gene expression. Cell-specific coexpression is determined by fitting regression models to each cell's k-nearest neighbours. A significant technical challenge we face is the sparsity, noise, and immense size of scRNA-seq data, which can lead to inaccurate results and make the analysis non-scalable. To address this challenge, we propose a solution using PC regression.

S3.3.1 PC regression

Most existing statistical gene network inference methods calculate gene coexpression directly within the gene expression space, involving computations across tens of thousands of gene pairs (Langfelder and Horvath, 2008; Huynh-Thu et al., 2010; Meyer et al., 2007). Performing this for each cell's neighbourhood is computationally infeasible.

PC regression is an attractive solution to the computational challenge we face (Jolliffe, 1982). Instead of fitting responses directly on gene expressions, we fit on the PC of gene expression. PCA not only speeds up model fitting by embedding data onto a much lower dimension, but also internally denoises and imputes scRNA-seq since a low-rank approximation (LRA) of the data can be recovered from PC. We derive gene-level importance of a PC regression model by estimating permutation feature importance (Section S3.3.2). In order to perform PC regression, we first run PCA on the full data including every cells

PCA Let $\mathcal{X}$ be a $N \times P$ (cell-by-gene) scRNA-seq gene expression matrix that is centered to zero mean and scaled to a unit variance. We extract the first $R < min(N, P)$ singluar vector of $\mathcal{X}$ to construct a rank-R LRA of $\mathcal{X}$ as

$$\widehat{\mathcal{X}} = \mathcal{U}\Lambda\mathcal{V}^T \tag{1}$$

Here, the $N \times R$ matrix $\mathcal{S} = \mathcal{U}\Lambda$ is known as the scores, and the $P \times R$ matrix $\mathcal{V}$ is known as the loading vectors in PCA.

We choose R according to the method described in (Linderman et al., 2022), who estimates

R by evaluating the spacing between singular values $s_r = \lambda_{r+1} - \lambda_r$, where $\lambda_r = \Lambda_{rr}$. R is chosen as the largest $r \in \{1, ..., 100\}$ such that s_r is smaller than a specific threshold determined by the distribution of $\{s_r : r \in \{1, ..., 100\}\}$. See the original paper for details.

Construct KNN-graph We begin by constructing a KNN graph of cells based on the distances between them in the PCA space. This KNN graph serves multiple purposes: it facilitates the execution of weighted PC regression, enables the calculation of weighted local variance of gene expression (as described in Section S3.3.2), and assists in denoising the coexpression estimation (detailed in Section S3.3.4).

The KNN-graph is constructed according to the uniform manifold approximation and projection algorithm, as outlined by Becht et al. (2019). Below, we describe the $N \times N$ weighted adjancency matrix $\mathcal{W}$ that represents the KNN-graph. The detailed reasoning for choosing this KNN-graph is similar to that discussed in (Deng et al., 2022), and we omit it here for brevity.

In the KNN graph, each cell $n \in \{1, \ldots, N\}$ is connected to its K-nearest neighbours, represented as a set $\text{KNN}(n) \subseteq \{1, \ldots, N\}$. The (n, m) entry of $\mathcal{W}$, which represents the weight of the edge directed from cell n to cell m on the KNN graph, is given by:

$$\mathcal{W}_{nm} = \begin{cases} \exp\left(-\frac{\max(0, d_{nm} - d_{n(2)})}{\delta_n}\right) & \text{if } m \in \text{KNN}(n) \\ 0 & \text{otherwise} \end{cases} \tag{2}$$

where $d_{nm} = \|\mathcal{S}_{n.} - \mathcal{S}_{m.}\|$ is the Euclidean distance in PC scores between cells n and m. The notation $d_{n(k)}$ denotes the distance from cell n to its kth nearest neighbour. Specifically, $k = 1$ refers to cell n itself, while $k = 2$ refers to the nearest neighbour of cell n. The scaling factor δ_n is chosen such that each node has a fixed out degree equals to $\log_2(K)$,

$$\sum_{j \in \text{KNN}(n)} \mathcal{W}_{nm} = \log_2(K) \tag{3}$$

In the following sections, we will use $\mathcal{W}_{n(k)}$ to denote the edge weight between cell n and its kth nearest neighbour, similar to the notation of $d_{n(k)}$.

PC regression We describe PC regression in cell n's K-nearest neighbourhood. To simplify notations, we use bold letter $(\mathbf{X}, \widehat{\mathbf{X}}, \mathbf{S})$ to distinguish local data from that of global ones $(\mathcal{X}, \widehat{\mathcal{X}}, \mathcal{S})$ respectively, without mentioning n.

Let $(\mathbf{X}, \widehat{\mathbf{X}})$ be $K \times P$ gene expression data sampled at cell n's K-nearest neighbourhood. The $K \times R$ matrix $\mathbf{S}$ and the length K vector $\widehat{y} = \widehat{\mathbf{X}}_{.p}$ represents the PC scores and the response p, respectively, for the cell neighbourhood. $\widehat{y}_{(k)}$ and $\mathbf{S}_{(k).}$ for $k \in \{1, ..., K\}$ represents the response and the PC score of the kth nearest neighbour of cell n respectively.

We weight cells in the neighbourhood of cell n according to the KNN-graph we constructed in the privous section. PC regression with data of the kth neighbour $(\widehat{y}_{(k)}, \mathbf{S}_{(k).})$ weighted by $\mathcal{W}_{n(k)}$ is then performed,

$$\widehat{y} \sim \mathbf{1}_K \widehat{y}_{(1)} + f_{np}(\mathbf{S} - \mathbf{1}_K \mathbf{S}_{(1).}) \tag{4}$$

where f_{np} is a regression model of choice. Since we are only interested in the local partial effect of PCs or genes on the response at cell n, we center the regression to cell n by adding a intercept term $\widehat{y}_{(1)}$ and centering $\mathbf{S}$ by $\mathbf{S}_{(1).}$. Our default choice of regression model is ridge regression with a penalty parameter set to 0.5. The solution of this ridge regression is equivalent to the maximum a posteriori solution of a Bayesian regression with standard normal priors on the regression coefficients, which is a prior choice suggested by (Bürkner, 2017).

S3.3.2 Permutation feature importance as coexpression measure

We first introduce the feature importance score for predictor gene q on response gene p, at cell n, denoted as $\mathcal{A}_{npq}$. This score is based on an approximation of permutation feature importance from eq. (12) and adjusted for bias in PC regression. By construction, $\mathcal{A}_{npq}$ is the square of the effect measure $\mathcal{B}_{npq}$, which can be intepreted as the change in the response gene expression resulting from a standard unit change in the predictor gene expression.

$$\mathcal{B}_{npq} = \sqrt{2}\,\mathrm{sd}(\mathbf{X}_{.q}) \left(\sum_r \hat{\beta}_{npr} \frac{\mathcal{V}_{qr}}{\|\mathcal{V}_{q.}\|} \right) \tag{5}$$

and

$$\mathcal{A}_{npq} = \mathcal{B}_{npq}^2 \tag{6}$$

Here, $\mathrm{sd}(\mathbf{X}_{.q})$ is the local weighted standard deviation of the scaled predictor gene q, weighted by $\mathcal{W}_{n(k)}$. The term $\hat{\beta}_{npr}$ is the partial derivative of the fitted regression function $\widehat{f_{np}}$ with respect to the rth principal component at cell n. In practice, our method returns $\mathcal{B}_{npq}$ instead of $\mathcal{A}_{npq}$ because the former retains the sign of the effect, providing more informative results. $\mathcal{A}_{npq}$ can be easily derived from $\mathcal{B}_{npq}$ when needed

The following sections detail the derivation and rationale behind our feature importance score.

Permutation feature importance We propose to measure coexpression between a response and predictor genes by the importance of predictor genes in fitting regression model. Since we do not want to impose parametric assumption on f, we consider using permutation feature importance, a commonly used predictor importance score in non-parametric regression. In our PC regression context, predictor q's importance is calculated by empirically estimating the following expectation

$$\mathcal{A}_{npq} = \mathbb{E}\left[\left\|\widehat{f_{np}}(\mathbf{S} - \mathbf{1}_K\mathbf{S}_{(1).}) - \widehat{f_{np}}(\mathbf{S}^{(q)} - \mathbf{1}_K\mathbf{S}_{(1).})\right\|^2\right] \tag{7}$$

where $\mathbf{S}^{(q)} = \widehat{\mathbf{X}}^{(q)}\mathcal{V}$ is the PC score projection with the qth gene of $\widehat{\mathbf{X}}$ being resampled locally in the cell neighbourhood. $\mathcal{A}_{npq}$ represents the mean squared difference between the original PC regression prediction, and the prediction made subject to resampling of gene q. A larger value of $\mathcal{A}_{npq}$ indicates that the gene is more influential in the regression model. Typically, $\mathcal{A}_{npq}$ is estimated by permuting the gene data and calculating the squared differences for multiple times, then averaging these differences over all permutations.

A simplified approach for estimation Permutation approach for estimating $\mathcal{A}_{npq}$ is computationally expensive, especially when we need to fit regression and permute for each gene in each cell's neighbourhood. To address this, we propose a simplified approach for estimating $\mathcal{A}_{npq}$. We numerically validated our proposed estimator on different PC regression models.

Let $\mathbf{X}$ be samples from a random vector X of length P, representing the distribution of gene

expression in the cell neighbourhood. Define $X^{(q)}$ as a variant of X, with $X_p^{(q)} = X_p$ for all $p \neq q$. $X_q^{(q)}$ is sampled from the same distribution as X_q, but independent from X_q. eq. (7) in this population setting can be simplified as

$$\mathcal{A}_{npq} = \mathbb{E}\left[\left(\widehat{f}_{np}(S - c) - \widehat{f}_{np}(S^{(q)} - c)\right)^2\right] \tag{8}$$

where $S = X\mathcal{V}$ and $S^{(q)} = X^{(q)}\mathcal{V}$ are length R random vectors representing local PC score distributions, c is a constant centering vector. Using a first-order Taylor expansion around 0, we obtain

$$\begin{aligned}
\mathcal{A}_{npq} &= \mathbb{E}\left[\left(\sum_r \frac{d\widehat{f}_{np}(s)}{ds_r}\Big|_{s=0}(S_r - S_r^{(q)})\right)^2\right] \\
&= \mathbb{E}\left[(X_q - X_q^{(q)})^2\left(\sum_r \frac{d\widehat{f}_{np}(s)}{ds_r}\Big|_{s=0}\mathcal{V}_{qr}\right)^2\right] \qquad (9) \\
&= \mathbb{E}\left[(X_q - X_q^{(q)})^2\right]\left(\sum_r \frac{d\widehat{f}_{np}(s)}{ds_r}\Big|_{s=0}\mathcal{V}_{qr}\right)^2 \qquad (10)
\end{aligned}$$

When centreing regression around cell n, $\frac{d\widehat{f}_{np}(s)}{ds_r}\Big|_{s=0}$ represents the partial derivative of $\widehat{f}_{np}(s)$ evaluated at cell n since $s = S - c = 0$. We simplify the notation of these partial derivative as $\hat{\beta}_{npr}$. In the case of a simple linear regression model, the derivative $\hat{\beta}_{npr}$ is the regression coefficient fitted on the rth component. In the cases of non-linear regressions, $\hat{\beta}_{npr}$ is estimated using symmetric derivative of moving average smoothed $\widehat{f}_{np}$ (Aull, 1967; Jacoby, 2000).

For calculating the first expectation term of eq. (10), we leverage the iid assumption of X_q and $X_q^{(q)}$ to derive

$$\mathbb{E}\left[(X_q - X_q^{(q)})^2\right] = 2\,\mathrm{Var}(X_q) \tag{11}$$

This expression represents twice the local variance of gene q, which can be computed directly without the need for permutations. Thus, the expression for $\mathcal{A}_{npq}$ becomes:

$$\mathcal{A}_{npq} = 2\,\mathrm{Var}(X_q)\left(\sum_r \hat{\beta}_{npr}\mathcal{V}_{qr}\right)^2 \tag{12}$$

$\mathcal{A}_{npq}$ can now be easily interpreted as we introduced at the beginning of Section S3.3.2, hence provides a straightforward measure of feature importance.

To evaluate the accuracy of the approximation in eq. (12) for $\mathcal{A}_{npq}$, we compared it against values of $\mathcal{A}_{npq}$ obtained via actual permutation, as defined in eq. (7) (Section S1.1). For linear models, including linear support vector machines and ridge regression, we achieved nearly perfect approximations. In the case of non-linear models like random forests, which have been successful in inferring gene coexpression (Huynh-Thu et al., 2010), we observed correlations exceeding 0.5 between results of approximation and permutation.

Adjust bias in PC regression Note that $\sum_r \hat{\beta}_{npr} \mathcal{V}_{qr}$ in eq. (12) represents the dot product between $\hat{\beta}_{np.}$ and $\mathcal{V}_{q.}$. This dot product is proportional to $\|\mathcal{V}_{q.}\|$, which is the magnitude of the loadings for gene q. Consequently, genes that are more influential in the PCA tend to receive higher importance scores. However, we contend that $\|\mathcal{V}_{q.}\|$ should not significantly influence the importance score. What truly matters is whether $\hat{\beta}_{np.}$ and $\mathcal{V}_{q.}$ are aligned in the same direction.

To address this bias, we propose scaling $\mathcal{V}_{q.}$ to a unit length. This adjustment leads to our final expression for the sample-level importance score, as proposed in eq. (5).

S3.3.3 Matrix representation of networks and network tensor

When performing regression on $P' \leq P$ response variables, we obtain a $P' \times P$ importance score matrix $\mathcal{A}_{n..}$, for each cell $n \in 1, \ldots, N$. This matrix $\mathcal{A}_{n..}$ serves as a representation of the coexpression network for cell n, where each element reflects the strength of the connection (i.e., coexpression calculated as described in eq. (5)) between pairs of nodes (i.e, genes).

Building on this concept, the $N \times P' \times P$ data entity $\mathcal{A}$, known as a network tensor, is a collection of the coexpression networks across all cells. Similarly, $\mathcal{B}$, which represents a stack of effect matrices, is referred to as an effect tensor.

S3.3.4 Graph denoise by random walk diffusion

Regression can be unstable and noisy when there is a small sample size in each cell's neighbourhood. Suppose we construct an $N \times P' \times P$ effect tensor $\mathcal{B}$. To denoise the effect estimation, we smooth

$\mathcal{B}$ over the KNN-graph of cells that is described in Section S3.3. We first symmetrise $\mathcal{W}$ as done by (Becht et al., 2019)

$$\mathcal{W}^{\text{sym}} = \mathcal{W} + \mathcal{W}^T - \mathcal{W} \circ \mathcal{W}^T \tag{13}$$

where $\circ$ denotes the Hadamard product. Next, we construct a random walk operator:

$$\mathcal{W}^{\text{rw}} = 0.5\mathcal{I} + 0.5\mathcal{D}^{-\frac{1}{2}}\mathcal{W}^{\text{sym}}\mathcal{D}^{-\frac{1}{2}} \tag{14}$$

where $\mathcal{I}$ is an identity matrix, $\mathcal{D}$ is a diagonal matrix with $D_{nn} = \sum_m \mathcal{W}_{nm}^{\text{sym}}$. The largest eigenvalue of $\mathcal{W}^{\text{rw}}$ is equal to 1, and the remaining eigenvalues are bounded by 0 and 1. This construction ensures that the diffusion process does not drift the scale of $\mathcal{B}$. The effect matrix of cell n, $\mathcal{B}_{n..}$, is denoised by applying the random walk operator on $\mathcal{B}$:

$$\widehat{\mathcal{B}}_{n..} = \sum_m g(\mathcal{W}^{\text{rw}})_{nm}\mathcal{B}_{m..} \tag{15}$$

where g is a function on the eigenvalues of $\mathcal{W}^{\text{rw}}$, given by:

$$g(\mathcal{W}^{\text{rw}}) = \Gamma^t\mathcal{E}\mathcal{E}^T \tag{16}$$

Here, Γ are the eigenvalues and $\mathcal{E}$ are the corresponding eigenvectors of $\mathcal{W}^{\text{rw}}$. t is a hyper-parameter that controls the number of steps of the random walk. If Γ and $\mathcal{E}$ represents the complete eigensystem of $\mathcal{W}^{\text{rw}}$, we have $g(\mathcal{W}^{\text{rw}})$ equals to the tth power of $\mathcal{W}^{\text{rw}}$. In practice, we only construct an eigensystem with a rank much smaller than N, allowing the diffusion to be more efficiently computed in eq. (15) via matrix multiplication. Moreover, the use of a rank-reduced $g(\mathcal{W}^{\text{rw}})$ can be interpreted as applying low-pass filtering on $\mathcal{B}$ (Smola and Kondor, 2003), resulting in a smoother $\widehat{\mathcal{B}}$ over the graph.

The smoothed importance score $\widehat{\mathcal{A}}$ can be calculated as an element-wise square of $\widehat{\mathcal{B}}$.

S3.3.5 Network pruning

The denoised effect tensor $\widehat{\mathcal{B}}$ is fully dense and contains a substantial amounts of insignificant effects that do not represent true coexpression. To prevent these insignificant effects from confounding the downstream analysis, we prune $\widehat{\mathcal{B}}$ independently for each response gene by calculating the statistical significance of effects and potentially shrink non-significant effects to zero. We describe in here the pruning of effects of P predictor genes on a specific response gene p, which is represented by the effect matrix $\widehat{\mathcal{B}}_{.p.}$ of size $N \times P$. Two strategies are provided: the first is heuristic but computationally efficient. This approach was used to run all the analyses in this paper; the second is more statistically rigorous but computationally intensive.

Pruning strategy one: random permutation The pruning strategy is based on the premise that response genes can act as their own predictors in the PC regression model. Given that a gene must be a causal regulator of itself, the effect of the response on itself, denoted by $\widehat{\mathcal{B}}_{.pp'}$, where p' is the index of the response as a predictor, provides a benchmark. This benchmark can identify effects that might indicate functional co-regulation with the response.

Specifically, let $\mathcal{B}_{.p.}$ represent the noisy coexpression with the response p and $\widehat{\mathcal{B}}_{.p.}$ the estimated signals derived from $\mathcal{B}_{.p.}$. To establish a null distribution of signals, we conduct random diffusion by permuting the entries (cells) in $\mathcal{B}_{.pp'}$ to create a vector of destructed effects, denoted by ω_p Picart-Armada et al. (2021). Subsequently, we apply the diffusion operator $g(\mathcal{W}^{\mathrm{rw}})$ as defined in eq. (15) to denoise ϵ_p:

$$\widehat{\epsilon}_p = g(\mathcal{W}^{\mathrm{rw}})\epsilon_p \tag{17}$$

The resulting vector $\widehat{\epsilon}_p$ represents the signals from random data, which we consider as indicative of insignificance. We repeat the permutation and calculate the mean $\widehat{\mu}_p$ and the standard deviation $\widehat{\sigma}_p$ of $\log|\widehat{\epsilon}_p|$ over permutation to establish the null effect distribution for p.

Pruning strategy two: hypothetical housekeeping gene A more statistically rigorous, though slightly more computationally intensive, approach prunes edges by benchmarking them

against a null distribution built from hypothetical housekeeping genes. Because all expression values are centred and scaled before PCA, a genuine housekeeping gene should display unit local variance and PC loadings that are symmetrically distributed around zero. Treating such a gene as pure Gaussian noise, we simulate its expected effect within each cell neighbourhood according to eq. 5 as:

$$\epsilon_{npq'} = \sqrt{2}\left(\sum_r \hat{\beta}_{npr}\frac{z_{q'r}}{\|z_{q'.}\|}\right) \tag{18}$$

where the pseudo-loading vector $z_{q'.}$ of the housekeeping gene q' is sampled from:

$$z_{q'r} \sim N\left(0, \frac{\nu_r}{(\nu_r - 1)^2}\right), \nu_r = \text{Global variance of PC } r \tag{19}$$

This distribution represents the first-order eigenvector-perturbation law for the loading of a standard Gaussian noise variable appended to the scaled data matrix. Subsequently, we apply the diffusion operator $g(\mathcal{W}^{\mathrm{rw}})$ as defined in eq. (15) to denoise $\epsilon_{.pq'}$:

$$\widehat{\epsilon}_{.pq'} = g(\mathcal{W}^{\mathrm{rw}})\epsilon_{.pq'} \tag{20}$$

We repeat the simulation, drawing an independent resample of $z_{q'.}$ each time. For gene p, we then pool all simulated effects and compute the mean $\widehat{\mu}_p$ and standard deviation $\widehat{\sigma}_p$ of the log-absolute effects, $\log|\widehat{\epsilon}_{.p.}|$, to establish the null effect distribution for p.

Significance calculation The significance of $\widehat{\mathcal{B}}_{npq}$, representing the effect of gene q in predicting the response in cell n, is given by

$$\Pi_{npq} = \Phi(\frac{\log|\widehat{\mathcal{B}}_{npq}| - \widehat{\mu}_p}{\widehat{\sigma}_p}) \tag{21}$$

Here, the left-hand side of the equation represents the lower tail probability of $\log|\widehat{\mathcal{B}}_{npq}|$ in a normal distribution $N(\widehat{\mu}_p, \widehat{\sigma}_p)$. In this context, Φ denotes the cumulative distribution function of the standard normal. We classify $\widehat{\mathcal{B}}_{npq}$ as significant if it deviates from the distribution of $\widehat{\epsilon}_p$, thereby receiving a larger value of Π_{npq} closer to 1.

A $N \times P' \times P$ tensor of significant scores Π is constructed by repeating the above procedure for each $p \in \{1, \ldots, P'\}$.

S3.4 Downstream analysis: embed network ensemble by non-negative PCA

After obtaining the denoised importance score tensor $\widehat{\mathcal{A}}$, which represents CSNs, an optional downstream analysis involves discovering the most informative network patterns in the data. This can be achieved by embedding the cell dimension using methods such as non-negative tensor factorisation (NTF).

However, given the potentially large size of $\widehat{\mathcal{A}}$, NTF may not be computationally feasible in terms of speed and memory usage. To address this, we simplify the procedure by applying non-negative matrix factorisation (NMF) to a matrix representation of $\widehat{\mathcal{A}}$. This is accomplished by vectorising the adjacency matrix of each cell, $\widehat{\mathcal{A}}_{n..}$, into a length PP' one-dimensional vector of edge weights a_n. The resulting tall-and-skinny matrix $\mathcal{A}^{\text{cell}} = [a_1, a_2, ..., a_N]$ should have $P'P >> N$ rows corresponding to edges between P' responses, and P predictors and N columns corresponding to cells. The NMF problem in general is formulated as

$$\|\mathcal{A}^{\text{cell}} - fh^T\|_F \quad \text{subject to} \quad h \geq 0$$

where f is a length PP' factor representing vectorised meta-network, and h is a length N loading representing N soft clusters.

We adapted the approach taken by Benson et al. (2014) to efficiently solve the NMF problem for tall-and-skinny matrices. At the core of this approach is the reduction of the row dimension of the matrix using singular value decomposition (SVD). Instead of applying NMF to the entire matrix, it is applied to the singular vectors obtained from SVD, which significantly reduces the computational complexity. We found that the approach is also valid when non-negative PCA (nPCA) is the NMF method.

We consider solving nPCA with the objective of maximising variances of PC scores subject to

the non-negativity constrain on loadings.

$$\underset{h}{\operatorname{argmax}}\, h^T \mathcal{M} h \quad \text{subject to} \quad \|h\| = 1, h \geq 0 \tag{22}$$

where the covariance matrix between cells $\mathcal{M}$ can be calculated by

$$\mathcal{M} = \sum_p \widehat{\mathcal{A}}_{.p.} \widehat{\mathcal{A}}^T_{.p.} \tag{23}$$

We observe that by diagnosing $\mathcal{M} = \mathcal{L}\Sigma\mathcal{L}$, we have

$$h^T \mathcal{M} h = h^T \mathcal{L}\Sigma\mathcal{L}^T h \tag{24}$$

Combining with eq. (24), a straightforward derivation shows that the objective in eq. (22) is equivalent to solving a restricted singular value decomposition (SVD) problem on $\Sigma^{\frac{1}{2}}\mathcal{L}^T$ (Shen and Huang, 2008), which has much lower dimension compared to the tall-and-skinny matrix previously described. Additionally, it eliminates the need to construct the tall-and-skinny matrix, thereby saving on extra memory usage. This SVD problem can be efficiently solved using iterative regression and deflation techniques, as detailed in Mackey (2008) and Sigg and Buhmann (2008). Without details in derivation, we describe the algorithm in below.

Algorithm 1 Non-negative principal component analysis (nPCA) on long and tall matrices

Require:

$\mathcal{Z} = \Sigma^{\frac{1}{2}}\mathcal{L}^T$ where Σ and $\mathcal{L}$ represent the eigenvalues and eigenvectors of the covariance matrix between cells $\mathcal{M}$ in eq. (23).

tol: numerical tolerance of the optimisation problem.

1: Create a $N \times N$ projection matrix $\boldsymbol{\Omega}$ that projects onto a space not spanned by the leading non-negative components. $\boldsymbol{\Omega}$ is initialised as an identity matrix. ▷ **Initialisation**

2: Sample a random length N vector h from uniform distribution. ▷ **Extract a single component**

3: $h \leftarrow h/\|h\|$

4: **while** TRUE **do**

5: $\quad h^{\text{old}} \leftarrow h$

6: $\quad z \leftarrow \mathcal{Z}h$

7: $\quad h \leftarrow \mathcal{Z}^T z$ ▷ **Regression**

8: $\quad$ Project h onto the positive quadrant such that $h_n \leftarrow \max(h_n, 0)$ for $n \in \{1, \dots, N\}$

9: $\quad h \leftarrow h/\|h\|$

10: $\quad$ **if** $\|h - h^{\text{old}}\| \leq \text{tol}$ **then**

11: $\quad\quad$ **Break;**

12: $h \leftarrow h/\|h^T \Omega h\|$

13: $h' \leftarrow \Omega h$ ▷ **Deflation**

14: $\mathcal{Z} \leftarrow \mathcal{Z}(\mathcal{I} - h'^T h')$

15: $\Omega \leftarrow \Omega(\mathcal{I} - h'^T h')$

16: $h \leftarrow h/\|h\|$

17: h is the resulting loading vector for a non-negative component. Repeat step 2 to 16 to extract subsequent components. ▷ **Multiple components**

To further improve computational efficiency in practice, we truncate $\Sigma^{\frac{1}{2}}\mathcal{L}^T$ by retaining only the leading eigenvalues in $\Sigma^{\frac{1}{2}}$. nPCA is performed on the truncated matrix.

S3.5 Downstream analysis: prior knowledge annotation of coexpression networks

Now we have a tensor $\widehat{\mathscr{A}}$ (see Section S3.3) that represents cell-specific coexpression networks. These networks are termed 'coexpression' because they indicate observed associations between gene expressions; however, they do not necessarily represent direct gene regulation. For instance, two genes may display highly correlated expression patterns without directly interacting with each other. Such coexpression can be confounded by common upstream regulators. To improve

the interpretability of our coexpression networks, our next task is to annotate them with prior knowledge regarding the direction of gene regulation. Genes that are correlated in expression and supported by prior evidence provide a more reliable representation of biologically relevant, context-specific regulatory mechanisms.

In this section, we first describe how we construct a confidential prior knowledge network (PKN) by integrating multiple gene regulation and signalling interaction databases using the R package `OmniPath` (Türei et al., 2021) (Section S3.5.1). We then explain how we incorporate this prior knowledge into our coexpression network (Section S3.5.2).

S3.5.1 Construct prior knowledge network with OmniPath

OmniPath is an R package that serves as an interface to an online database of prior knowledge in intra- and inter-cellular signalling interactions. It also provides pipelines for integrating user-chosen databases and quality controls to build custom PKN. We build our network with OmniPath version 3.8.2, and adapted the built-in NicheNet integration pipeline originally proposed by Browaeys et al. (2020).

Network resources We run `OmniPath::nichenet_networks` to extract public inter- and intra-cellular signalling networks, and gene regulatory networks. We only consider direct interaction networks curated by Omnipath. For inter-cellular signalling networks, we only consider ligands and secreted enzymes as parental nodes, with receptors and transporters as child nodes. For the gene regulatory network, we exclusively use the CollecTRI database, which is an integrated database of high-quality evidence for TF and target interactions (Müller-Dott et al., 2023).

NicheNet Prior Knowledge Integration with Omnipath NicheNet is a popular method that models ligand and target gene interactions using prior knowledge networks Browaeys et al. (2020). In the paper, the authors describe a method to integrate networks from different sources using automatic parameter optimization, with a objective of building an integrated network that best aligns with ligand perturbation experiments. We implement NicheNet integration with Omnipath, following the tutorial at `https://r.omnipathdb.org/articles/nichenet.html` (Last retrieved:

July 30th, 2024).

The integration process yields a weighted gene regulatory network, a weighted signalling interaction network that encompasses both inter- and intra-cellular signalling pathways, and a matrix summarising ligands' regulatory potential on the other genes. These integrated data sets are provided by our package and are readily available for user application.

Receptor-target regulatory potential After the NicheNet integration, a ligand regulaory potential matrix is constructed by applying the personalized PageRank (PPR) algorithm to the signalling interaction PKN (Page, 1999). Specifically, the regulatory potential computed by PPR represents the probability that a signal, originating from a ligand, is transmitted to the target in the steady state of a random walk on the PKN.

In a similar manner, we construct a receptor regulatory potential matrix. We implement PPR using `igraph::page_rank` function from the R package `igraph` (version 2.0.1.1) (Csardi and Nepusz, 2006). Other than setting receptors as the source nodes, the parameters for running PPR are the same as those optimized during the integration process for the ligand regulaory potential matrix.

S3.5.2 Prior knowledge annotation

Here, we focus on the annotation for coexpression networks between TFs and potential target genes. In this context, one set (either TFs or targets) as responses and the other as predictors. The underlying assumption is that gene regulation requires coexpression between TFs and targets. In contrast, receptors do not need to be coexpressed with their targets to transmit signals; they only need to be expressed.

Annotate Co-Expression Networks We categorise edges (i.e., coexpression) in $\widehat{\mathcal{A}}$ into four confidence levels. These levels will be visually distinguished in the network visualisation function provided by our package. The four confidence levels are described in an increasing order below:

- Non-significantly coexpressed: The significance of an edge is calculated according to S3.3.2. These edges are not be shown in the visualisation.

- Significantly coexpressed, but lacking evidence of physical interaction: These edges are classified as significant, but there is no existing evidence in the prior gene regulatory network to support that the coexpression is regulatory. These edges are represented as dashed lines in the coexpression network.

- Significantly coexpressed and interacting within a few steps of signal transduction: These edges are classified as significant, and the TF signal can reach the target within a few steps (as user defined) in the prior gene regulatory network. These edges are represented as indirected solid lines in the coexpression network.

- Significantly coexpressed and directly interacts: These edges are classified as significant, and the corresponding TF directly interact with the target (in one step) in the prior gene regulatory network. These edges are represented as directed solid arrows in the coexpression network. Activation and suppression regulation are distinguished by the arrow heads.

Infer potential upstream signalling pathways of target genes We describe a scenario where we run KNN-PC regression using target genes as responses, and aim to infer upstream signalling pathways or receptors potentially affecting target expression in a context-specific manner. We begin by measuring receptor activity on the targets by combining coexpression networks with a prior model of receptor potential, created using the PPR algorithm (see Section S3.5.1). A receptor i's activity in regulating a single target p in cell n is quantified by

$$\sum_{q \in \{\text{TFs in predictors}\}} \mathbf{PPR}_{iq} \cdot \widehat{\mathcal{A}}_{npq} \cdot \mathbb{1}\{\widehat{\mathcal{A}}_{npq} \text{ passes a user defined confidence level}\} \tag{25}$$

where $\mathbf{PPR}_{iq}$ represents the receptor's regulatory potential on TF q. Summand in the equation reflects the receptor's inverse regulatory distance to the target, mediated by TFs, and is further pruned by the confidence of $\widehat{\mathcal{A}}_{npq}$.

For multiple targets, we can perform integrative analysis between the receptor potential matrix **PPR** and the cell n's coexpression network $\widehat{\mathcal{A}}_{n..}$, using tools such as those in the mixOmics package (Rohart et al., 2017). We describe a partial least square regression (PLS) (Wold et al., 2001)

approach that is a multivariate analogue to eq. (25). We first extract relevant data seubsets $\mathbf{PPR}^{\mathrm{TF}}$ and $\widehat{\mathcal{A}}_{n..}^{\mathrm{TF}}$ that have matching columns that represents known TFs in the predictors. The PLS algorithm finds linear embeddings for receptors and targets that maximize the covariance of TFs in this embedded space:

$$\underset{a,b}{\operatorname{argmax}} \ \operatorname{Cov}(a^T \mathbf{PPR}^{\mathrm{TF}}, b^T \widehat{\mathcal{A}}_{n..}^{\mathrm{TF}}) \quad \text{subject to} \quad \|a\| = \|b\| = 1 \tag{26}$$

Intuitively, a and b represent receptor and target modules that are most strongly connected through TFs. Receptors that are highly weighted by a are more likely to actively regulate the targets.

Finally, we infer the potential signalling pathway between a likely active receptor i and a target p. To do this, we first identify the TF q through which the distance between the receptor and the target is the shortest. Specifically, this TF is found by

$$\underset{q \in \{\text{TFs in predictors}\}}{\operatorname{argmax}} \quad \mathbf{PPR}_{iq} \cdot \widehat{\mathcal{A}}_{npq} \cdot \mathbb{1}\{\widehat{\mathcal{A}}_{npq} \text{ passes a user defined confidence level}\} \tag{27}$$

We simply consider the potential signalling pathway as the shortest path between TF q and the receptor, which is searched on the prior signalling network by the R igraph function `igraph::shortest_paths`.

S3.6 Analysis on subsampled data

Up to now, we have presented a computationally efficient algorithm for inferring cell-specific coexpression networks. However, we have not yet addressed the issue of memory usage. Storing dense networks for tens of thousands of cells is impractical on a personal computer and can be costly on a server.

We propose to address the memory usage problem by implementing parts of our pipeline on only a subset of cells that are representative of the full dataset. The following steps are performed on the entire dataset:

- PCA, LRA and KNN-graph construction.

For the representative subset of cells, we conduct:

- PC regression, importance score calculation, graph denoise and nPCA to construct meta-network.

Select representative cells To select representative cells, we perform k-means clustering on the PC scores S with N' centers. Within each cluster, we choose the cells most similar to the cluster center as representative cells for that cluster. Other sophisticated method, such as MetaCell (Baran et al., 2019), for selecting representative cells can be integrated seamlessly into our method. Next we describe the analysis on representative cells in more details.

PC regression and importance score calculation For a representative cell, its neighbours, the weighting of neighbours, and the regression data are consistent with the full analysis. Thus, the procedure is equivalent to applying the methods described in Sections S3.3.1 and S3.3.2 for each cell $n \in 1, \ldots, N$, while skipping those non-representative cells. Ultimately, this process yields a smaller effect tensor $\mathcal{B}'$ of size $N' \times P' \times P$ that is a subset of $\mathcal{B}$ obatined from the full analysis.

Graph denoise We denoise $\mathcal{B}'$ and, if needed, impute the effects of non-representative cells using network propagation. First, we construct an $N \times N'$ weight matrix $\mathcal{W}'$, which extracts columns of $g(\mathcal{W}^{\mathrm{rw}})$ defined in eq. (16), corresponding to the representative cells in $\mathcal{B}'$. We then further normalise $\mathcal{W}'$ by rows so that each row sums to one. The denoised (or impute) effect of cell n $\widehat{\mathcal{B}}'_{n..}$ for $n \in \{1, \ldots, N\}$ is computed by

$$\widehat{\mathcal{B}}'_{n..} = \sum_{m}^{N'} \mathcal{B}'_{m..} \mathcal{W}'_{nm} \tag{28}$$

Network pruning The null distribution of effect size for a response p is learned analogously as in S3.3.5 using $\mathcal{B}'_{.pp'}$, which is the response's effect on itself across representative cells. Cells, including non-representing ones, are pruned by comparing $\widehat{\mathcal{B}}_{.p.}$ with the null distribution, as done in eq. (21).

nPCA nPCA is conducted on the denoised importance score tensor of representative cells.

References

Aull, C. E. (1967). The first symmetric derivative. The American Mathematical Monthly, 74(6):708–711.

Baran, Y., Bercovich, A., Sebe-Pedros, A., Lubling, Y., Giladi, A., Chomsky, E., Meir, Z., Hoichman, M., Lifshitz, A., and Tanay, A. (2019). Metacell: analysis of single-cell rna-seq data using k-nn graph partitions. Genome biology, 20:1–19.

Becht, E., McInnes, L., Healy, J., Dutertre, C.-A., Kwok, I. W., Ng, L. G., Ginhoux, F., and Newell, E. W. (2019). Dimensionality reduction for visualizing single-cell data using umap. Nature biotechnology, 37(1):38–44.

Benson, A. R., Lee, J. D., Rajwa, B., and Gleich, D. F. (2014). Scalable methods for nonnegative matrix factorizations of near-separable tall-and-skinny matrices. Advances in neural information processing systems, 27.

Browaeys, R., Saelens, W., and Saeys, Y. (2020). Nichenet: modeling intercellular communication by linking ligands to target genes. Nature methods, 17(2):159–162.

Bürkner, P.-C. (2017). brms: An R package for Bayesian multilevel models using Stan. Journal of Statistical Software, 80(1):1–28.

Chan, J. M., Quintanal-Villalonga, A., Gao, V. R., Xie, Y., Allaj, V., Chaudhary, O., Masilionis, I., Egger, J., Chow, A., Walle, T., et al. (2021). Signatures of plasticity, metastasis, and immunosuppression in an atlas of human small cell lung cancer. Cancer cell, 39(11):1479–1496.

Csardi, G. and Nepusz, T. (2006). The igraph software. Complex syst, 1695:1–9.

Dai, H., Li, L., Zeng, T., and Chen, L. (2019). Cell-specific network constructed by single-cell rna sequencing data. Nucleic acids research, 47(11):e62–e62.

Deng, Y., Choi, J., and Lê Cao, K.-A. (2022). Sincast: a computational framework to predict cell identities in single-cell transcriptomes using bulk atlases as references. Briefings in Bioinformatics, 23(3):bbac088.

Dixit, A., Parnas, O., Li, B., Chen, J., Fulco, C. P., Jerby-Arnon, L., Marjanovic, N. D., Dionne, D., Burks, T., Raychowdhury, R., et al. (2016). Perturb-seq: dissecting molecular circuits with scalable single-cell rna profiling of pooled genetic screens. cell, 167(7):1853–1866.

Holland, C. H., Tanevski, J., Perales-Patón, J., Gleixner, J., Kumar, M. P., Mereu, E., Joughin, B. A., Stegle, O., Lauffenburger, D. A., Heyn, H., et al. (2020). Robustness and applicability of transcription factor and pathway analysis tools on single-cell rna-seq data. Genome biology, 21:1–19.

Huynh-Thu, V. A., Irrthum, A., Wehenkel, L., and Geurts, P. (2010). Inferring regulatory networks from expression data using tree-based methods. PloS one, 5(9):e12776.

Jacoby, W. G. (2000). Loess:: a nonparametric, graphical tool for depicting relationships between variables. Electoral studies, 19(4):577–613.

Jolliffe, I. T. (1982). A note on the use of principal components in regression. Journal of the Royal Statistical Society Series C: Applied Statistics, 31(3):300–303.

Langfelder, P. and Horvath, S. (2008). Wgcna: an r package for weighted correlation network analysis. BMC bioinformatics, 9(1):1–13.

Linderman, G. C., Zhao, J., Roulis, M., Bielecki, P., Flavell, R. A., Nadler, B., and Kluger, Y. (2022). Zero-preserving imputation of single-cell rna-seq data. Nature communications, 13(1):192.

Mackey, L. (2008). Deflation methods for sparse pca. Advances in neural information processing systems, 21.

Meyer, P. E., Kontos, K., Lafitte, F., and Bontempi, G. (2007). Information-theoretic inference of large transcriptional regulatory networks. EURASIP journal on bioinformatics and systems biology, 2007:1–9.

Müller-Dott, S., Tsirvouli, E., Vazquez, M., Ramirez Flores, R. O., Badia-i Mompel, P., Fallegger, R., Türei, D., Lægreid, A., and Saez-Rodriguez, J. (2023). Expanding the coverage of regulons

from high-confidence prior knowledge for accurate estimation of transcription factor activities. Nucleic acids research, 51(20):10934–10949.

Page, L. (1999). The pagerank citation ranking: Bringing order to the web. Technical report, Technical Report.

Papalexi, E., Mimitou, E. P., Butler, A. W., Foster, S., Bracken, B., Mauck III, W. M., Wessels, H.-H., Hao, Y., Yeung, B. Z., Smibert, P., et al. (2021). Characterizing the molecular regulation of inhibitory immune checkpoints with multimodal single-cell screens. Nature genetics, 53(3):322–331.

Picart-Armada, S., Thompson, W. K., Buil, A., and Perera-Lluna, A. (2021). The effect of statistical normalization on network propagation scores. Bioinformatics, 37(6):845–852.

Rohart, F., Gautier, B., Singh, A., and Lê Cao, K.-A. (2017). mixomics: An r package for 'omics feature selection and multiple data integration. PLoS computational biology, 13(11):e1005752.

Shen, H. and Huang, J. Z. (2008). Sparse principal component analysis via regularized low rank matrix approximation. Journal of multivariate analysis, 99(6):1015–1034.

Sigg, C. D. and Buhmann, J. M. (2008). Expectation-maximization for sparse and non-negative pca. In Proceedings of the 25th international conference on Machine learning, pages 960–967.

Smola, A. J. and Kondor, R. (2003). Kernels and regularization on graphs. In Learning Theory and Kernel Machines: 16th Annual Conference on Learning Theory and 7th Kernel Workshop, COLT/Kernel 2003, Washington, DC, USA, August 24-27, 2003. Proceedings, pages 144–158. Springer.

Tian, L., Dong, X., Freytag, S., Lê Cao, K.-A., Su, S., JalalAbadi, A., Amann-Zalcenstein, D., Weber, T. S., Seidi, A., Jabbari, J. S., et al. (2019). Benchmarking single cell rna-sequencing analysis pipelines using mixture control experiments. Nature methods, 16(6):479–487.

Türei, D., Valdeolivas, A., Gul, L., Palacio-Escat, N., Klein, M., Ivanova, O., Ölbei, M., Gábor, A.,

Theis, F., Módos, D., et al. (2021). Integrated intra-and intercellular signaling knowledge for multicellular omics analysis. Molecular systems biology, 17(3):e9923.

Wang, X., Choi, D., and Roeder, K. (2021). Constructing local cell-specific networks from single-cell data. Proceedings of the National Academy of Sciences, 118(51):e2113178118.

Wold, S., Sjöström, M., and Eriksson, L. (2001). Pls-regression: a basic tool of chemometrics. Chemometrics and intelligent laboratory systems, 58(2):109–130.
